# Epithelial–Mesenchymal Transition in Cancer: Insights Into Therapeutic Targets and Clinical Implications

**DOI:** 10.1002/mco2.70333

**Published:** 2025-08-29

**Authors:** Dhasarathdev Srinivasan, Ranjith Balakrishnan, Ankush Chauhan, Jeevan Kumar, Dinesh Murugan Girija, Reena Shrestha, Rupendra Shrestha, Rajasekaran Subbarayan

**Affiliations:** ^1^ Centre for Advanced Biotherapeutics and Regenerative Medicine Faculty of Research Chettinad Hospital and Research Institute Chettinad Academy of Research and Education Kelambakkam Tamil Nadu India; ^2^ Centre for Herbal Pharmacology and Environmental Sustainability Chettinad Hospital and Research Institute Chettinad Academy of Research and Education Kelambakkam Tamil Nadu India; ^3^ Department of Biomedical Sciences The Apollo University Chittoor Andhra Pradesh India; ^4^ Vopec Pharmaceuticals Pvt. Limited Research and Development Division Chennai India; ^5^ Department of Internal Medicine Berkshire Medical Center Pittsfield Massachusetts USA; ^6^ Department of Natural and Applied Sciences Nexus Institute of Research and Innovation (NIRI) Lalitpur Nepal

**Keywords:** biomarkers, cancer therapy, epithelial–mesenchymal transition, radiation therapy, signaling pathways, tissue injury

## Abstract

Radiation therapy is a fundamental component of cancer treatment, benefiting 50%–70% of patients by selectively targeting malignant tissues. However, radioresistance remains a significant challenge, often driven by epithelial–mesenchymal transition (EMT). EMT increases cancer invasiveness and metastasis by upregulating mesenchymal markers, including vimentin and N‐cadherin, and downregulating epithelial markers, such as E‐cadherin. EMT under radiation involves principal signaling pathways, including TGF‐β, Wnt/β‐catenin, Notch, and ERK, which regulate EMT through transcription factors such as Snail, Slug, Twist, and Zeb1/2. These alterations drive cytoskeletal reorganization, decrease cell–cell adhesion, and enhance extracellular matrix degradation via integrins, MMP‐2, and MMP‐9. We also explored how growth hormones, inflammatory cytokines, and hypoxia in the tumor microenvironment affect radiation‐induced EMT. Targeting EMT pathways with monoclonal antibodies and small‐molecule inhibitors of signaling pathways may help overcome radioresistance. However, due to the dual role of EMT in cancer progression and tissue regeneration, precise treatment strategies are essential. There is a lack of comprehensive multi‐omics studies that provide insights into postradiation EMT progression. This review examines how radiation induces EMT and its impact on metastasis and immune responses while also proposing therapeutic approaches. Integrating EMT‐targeting strategies with existing cancer treatments could enhance the effectiveness of radiotherapy and improve patient outcomes.

## Graphical abstract

This graphical abstract summarizes the effects of ionizing radiation (IR) on epithelial–mesenchymal transition (EMT), emphasizing critical processes such as DNA damage repair, tumor microenvironment interactions, and resistance mechanisms. It highlights EMT‐induced radioresistance regulators, such as TGF‐β, Wnt, Notch, ERK, and various miRNAs, as well as therapeutic strategies, including immune checkpoint inhibitors, immune modulators, and cancer therapies. In addition, this diagram highlights the challenges in EMT research and treatment strategies aimed at overcoming radioresistance.

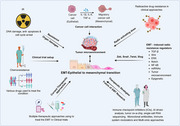

## 1 Introduction

Elizabeth Dexter “Betty” Hay (1927–2007), a pioneering cellular and developmental biologist at Harvard Medical School, was the first to describe epithelial–mesenchymal transition (EMT). Her foundational research began in 1958, focusing on amphibian limb regeneration, particularly the dedifferentiation of cartilage cells in salamanders, a process that resembles EMT. She later explored the role of the extracellular matrix (ECM) in epithelial cell differentiation, demonstrating that ECM composition influences corneal epithelial differentiation and the secretion of ECM proteins, such as collagen and glycosaminoglycans [1, 2]. In 1968, at the 18th Hahnemann Symposium, Hay elucidated the process by which epithelial cells differentiate into mesenchymal tissues during the migration of neural crest cells during neural tube development. Other researchers have observed the simultaneous presence of epithelial and mesenchymal cells within mixed uterine tumors [1, 3]. Hay's lab coined the term “epithelial to mesenchymal transformation” in 1982 while studying chicken lens epithelial cells cultured in collagen gels. These cells develop pseudopods and adopt a mesenchymal‐like morphology with migratory capacity [4]. Around this period, Dulbecco et al. also reported a “cuboid‐to‐fusiform transition” in rat mammary tumor cells, and other groups in Europe observed epithelial cells transforming into mesenchymal‐like cells during early embryogenesis, noting the loss of epithelial markers such as desmosomes and cytokeratin and the gain of mesenchymal traits such as vimentin expression [5].

Hay et al. advanced EMT research by incorporating molecular techniques into their studies. In 1986, using western blotting, they demonstrated that chicken lens epithelial cells undergoing EMT lost Type IV collagen and α‐crystallin while expressing Type I collagen [6]. Further studies on thyroid epithelial cells have revealed cytoskeletal remodeling, vimentin expression, and loss of thyroglobulin, indicating cellular dedifferentiation. Hay later proposed the “fixed cortex theory” to explain neural crest cell migration and emphasized the importance of cell‐matrix interactions in mesenchymal cell motility. The group also identified the role of EMT in embryonic palatal fusion [7]. In a 1990 publication, Hay discussed the importance of cell‐matrix interactions in mesenchymal cell migration [8]. Based on previous information, EMT is the primary biological process in which epithelial cells lose polarity and intercellular adhesion to acquire mesenchymal features, including enhanced motility, invasiveness, apoptosis resistance, and ECM production. It occurs in three processes: embryogenesis (Type I), tissue repair (Type II), and cancer progression (Type III) [9]. In cancer, EMT is driven by signaling pathways such as TGF‐β, Wnt/β‐catenin, ERK, Notch, and hypoxia‐related factors. These activate transcription factors such as Snail, Slug, Twist, and ZEB1/2, suppress epithelial markers such as E‐cadherin, and promote mesenchymal markers such as vimentin, N‐cadherin, fibronectin, and α‐smooth muscle actin. EMT contributes to tumor invasion, metastasis, resistance to therapy, immune evasion, and the acquisition of cancer stem cell (CSC) traits [10].

Approximately 50%–70% of cancer patients undergo radiotherapy (RT), but radioresistance, often linked to EMT, limits its success. Ionizing radiation can induce EMT via the TGF‐β, Notch, and ERK pathways, thereby promoting tumor cell plasticity, survival, and recurrence. Post‐RT, EMT marker shifts enable residual cells to invade and metastasize [11]. Despite these challenges, EMT presents an opportunity for targeted therapy. Inhibitors of TGF‐β, Wnt/β‐catenin, and Notch, ranging from small molecules to noncoding RNA modulators, have shown promise in preclinical models [12]. Recent advances have indicated promising clinical applications, such as EMT‐targeted therapies to inhibit tumor progression and metastasis, along with the use of EMT markers for early diagnosis and prognosis. We also discuss recent clinical trials around the world investigating the role of EMT in cancer progression and metastasis, as well as potential therapeutic strategies targeting EMT. This review has the potential to enhance our understanding and lead to new interventions for cancer and fibrotic diseases. Despite this progress, challenges persist in applying EMT to develop safe and effective therapies, underscoring the necessity for ongoing research in this field. Combining EMT inhibitors with existing therapies may improve patient outcomes. However, care must be taken to avoid disrupting the physiological EMT required for tissue repair and regeneration. The dynamic and information‐dependent nature of EMT underscores the need for novel approaches to cancer therapy. Understanding the intricate signaling networks and identifying reliable EMT biomarkers could facilitate early diagnosis, patient stratification, and the development of personalized treatment regimens. This review provides a comprehensive analysis of the molecular mechanisms underlying EMT, its role in cancer radioresistance, and recent advances in therapeutic strategies to mitigate EMT‐associated treatment failure. We focused on the most relevant signaling pathways, emerging therapeutic targets, and innovative EMT inhibitors, and discussed approaches to reduce radiation‐induced tissue damage.

## 2 EMTs in Cancer

**FIGURE 1:**
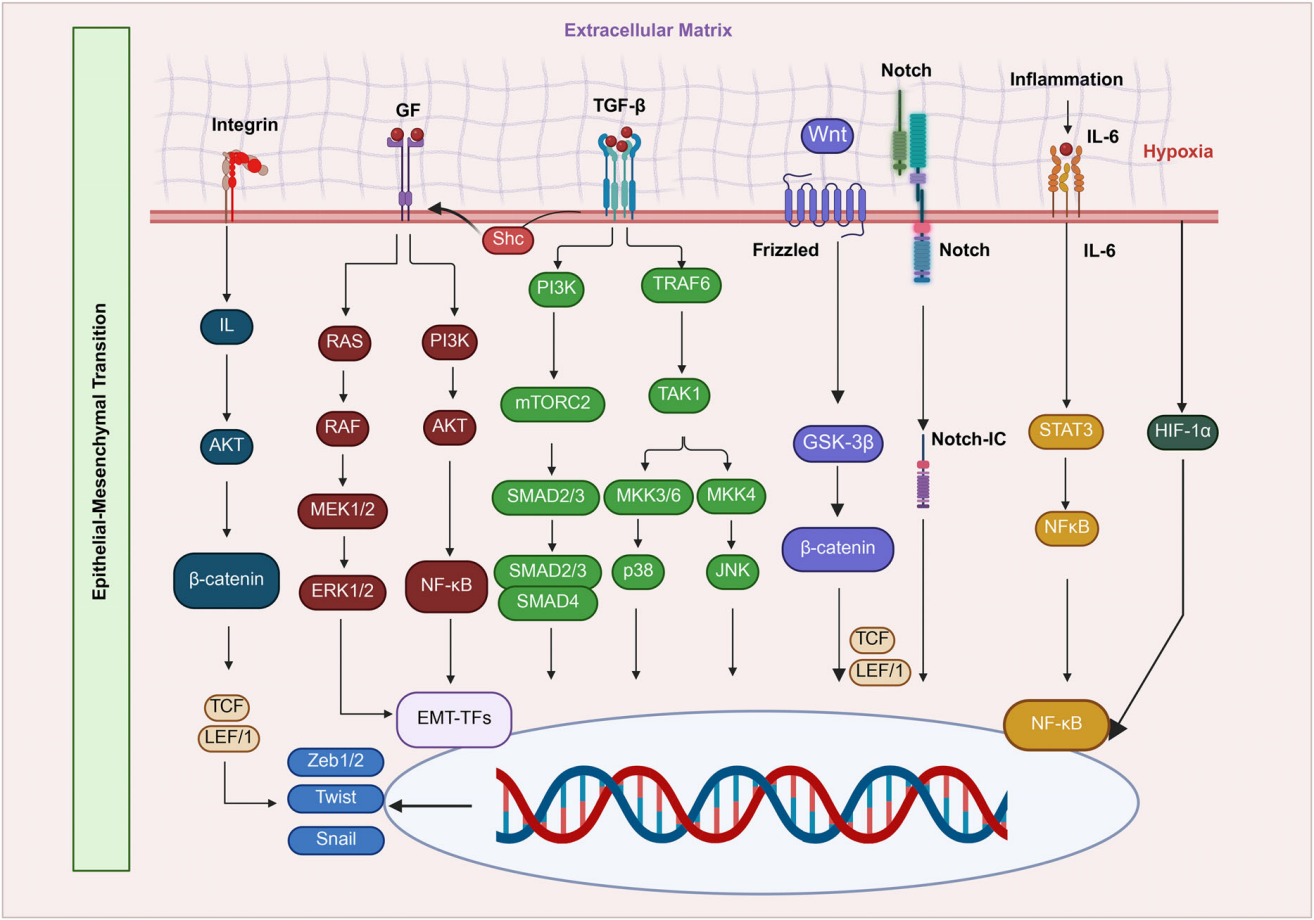
Schematic representation of the signaling pathways involved in EMT, development, tissue repair, and cancer metastasis. EMT is triggered by extracellular signals, including integrins for cell adhesion and growth factor (GF) receptors that activate downstream cascades. The TGF‐β pathway induces EMT, whereas the Wnt/Frizzled pathway regulates its signaling. Notch signaling and the inflammatory mediator IL‐6 also contribute to hypoxia, along with environmental factors that activate HIF1α. Several pathways converge intracellularly to regulate EMT. The PI3K/AKT pathway supports cell survival, whereas the RAS/RAF/MEK/ERK pathway promotes cell proliferation. The SMAD pathway, activated by TGF‐β, directly regulates the transcription of EMT‐related genes. Other pathways, including GSK‐3β/β‐catenin and various stress‐responsive pathways, further modulate EMT. These signaling cascades activate transcription factors (EMT‐TFs), such as Zeb1/2, Twist, and Snail, leading to the repression of epithelial markers, such as E‐cadherin, and promoting mesenchymal transition.

Cancer cells utilize the dynamic process of EMT to gain migratory and invasive characteristics, promote metastasis, and frequently develop treatment resistance [13]. EMT entails several alterations at the cellular and molecular levels, notably reorganization of the cytoskeleton and a reduction in cell–cell adhesion and polarity, which jointly augment cellular motility and invasiveness. EMT causes many changes at the cellular and molecular levels, especially cytoskeletal reconstitution of cell–cell adhesion and decreased polarity, which increases cellular motility and permeability [14]. Cancer cells employ EMT to acquire migratory and invasive traits, promote metastasis, and often develop resistance to treatment. EMT results in cytoskeletal rearrangement, reduced cell–cell adhesion, and increased cellular motility and invasiveness. EMT factors such as SNAIL, SLUG, ZEB1, and TWIST regulate E‐cadherin synthesis. Loss of polarity between the apex and base is crucial for tissue architecture and function, as it is maintained by epithelial cells [15]. Figure 1 shows the signaling pathways involved in EMT, which is a significant process in development, tissue repair, and cancer metastasis. During EMT, polarity is reversed, enabling cells to adopt a fibroblast‐like shape. The breakdown of the PAR and Crumbs complexes regulates this change. Disruptions in cell interactions and issues with adherence and tight junction proteins lead to a migratory phenotype [16]. N‐cadherin enhances weak cell–cell adhesion, facilitating the adaptable connections required for migration. The actin cytoskeleton, which is essential for cell structure, changes significantly during EMT [17]. Cofilin destabilizes actin filaments, aiding cytoskeletal reorganization, whereas profilin accelerates actin polymerization to synthesize more filaments as needed [18]. Cell migration relies on tensile and contractile forces generated by stress fibers, which enhance cell flexibility and mobility. Filopodia, characterized by their slender, spike‐like appearance, and lamellipodia, distinguished by their elongated, sheet‐like structures, are protrusions that develop on cancer cell surfaces due to EMT. Small signaling molecules such as RhoA, Rac1, and Cdc42 regulate cytoskeletal reorganization. Rac1 is crucial for lamellipodia production, allowing cell‐front extension, whereas Cdc42 promotes filopodia formation for cell exploration and interaction [19]. Directed movement is crucial for tissue migration, and protrusions interact with the ECM to facilitate this process. During EMT, integrin receptor levels increase, enhancing cell adhesion to ECM components such as collagen and fibronectin. ECM proteins are degraded by metalloproteinases (MMPs), particularly MMP‐2 and MMP‐9. These enzymes facilitate cellular invasion of tissues by destroying the ECM [20]. A critical phase of metastasis results in increased integrin expression and ECM degradation, enabling cancer cells to detach from their primary sites, migrate to adjacent tissues, and invade specific niches.

## 3 Radiation‐Induced EMT Mechanisms of Target Receptors

Radiation damage to normal tissues can have both acute and chronic effects. It immediately harms the parenchymal and vascular endothelial cells, leading to acute damage. Radiation‐induced free radicals and reactive oxygen species (ROS) cause a persistent pro‐inflammatory state and increase cytokine levels, such as TNF‐α, IFN‐γ, IL‐1, and IL‐6. It reduces the number of cells by selectively targeting cells in the G2 and M phases of the cell cycle [21]. Changes in the ECM lead to elevated levels of MMPs and dysregulated collagen production. The microvascular system also undergoes alterations. The formation of new blood vessels is slowed, whereas the development of fibrous tissue within blood vessels is intensified [22]. Radiation exposure can cause long‐term damage, leading to the blockage of small blood vessels and scar tissue formation. This interrupts the healing process and reduces oxygen supply to areas, impairing the body's wound‐healing system over time through various genetic and cellular changes [23]. A significant majority of cancer patients (85% and 95%) experience skin damage during treatment. Radiation fibrosis syndrome can severely affect the muscles, ligaments, and tendons, leading to weakness, spasms, and decreased flexibility and mobility [24]. Osteoradionecrosis greatly affects a patient's quality of life through pain, bleeding, and bone exposure. Radiation exposure damages bone tissue, leading to oxidative stress, inflammation, cellular depletion, ECM abnormalities, and microvascular changes [25]. Understanding the mechanisms underlying the mitigation of the harmful effects of radiation is thus crucial. Both short‐ and long‐term consequences damage tissues. By targeting signaling pathways related to EMT, such as TGF‐β, Notch, Wnt, and ERK, we can reverse EMT and enhance cellular resistance to radiation [26, 27]. Recent studies have shown that the Notch signaling system is crucial for radiation‐induced EMT in breast cancer cells. Inhibition of this signaling pathway may enhance the susceptibility of cancer cells to radiation therapy [28]. This involves the regulation of specific transcription factors linked to EMT, along with various transcription factors and signaling pathways that facilitate its occurrence. The main functions of miRNAs in EMT in different types of cancer and their significance in radiation therapy are listed in Table 1.

**TABLE 1:** Overview of organ‐specific miRNAs and their target receptors/proteins in EMT in animal models.

Organ	miRNA	Target receptors	Functions	Animal model	Ref.
**Liver**	miR‐122	Cyclin G1, ADAM17	Inhibits EMT and liver fibrosis by targeting Cyclin G1 and ADAM17 and maintains the epithelial phenotype.	Mouse model of liver cancer	[29]
	miR‐21	PTEN, TGF‐β	Enhances EMT and contributes to liver cancer progression by targeting PTEN and promoting TGF‐β signaling.	Mouse model of liver fibrosis	[30]
	miR‐221/222	TIMP3, PTEN	Promotes EMT and hepatocellular carcinoma progression by downregulating TIMP3 and PTEN.	Mouse model of liver cancer	[31]
	miR‐29b	Collagen, TGF‐β	Suppresses EMT and liver fibrosis by inhibiting collagen synthesis and TGF‐β signaling.	Rat model of liver fibrosis	[32]
**Heart**	miR‐34a	SNAIL1, SNAIL2	Inhibits EMT and reduces cardiac fibrosis by targeting SNAIL transcription factors.	Mouse model of cardiac fibrosis	[33]
	miR‐146a	TRAF6, IRAK1	Inhibits EMT, reducing inflammatory signaling and cardiac fibrosis.	Mouse model of cardiac fibrosis	[34]
	miR‐21	PTEN, TGF‐β	Promotes cardiac fibrosis by enhancing EMT through PTEN inhibition and TGF‐β activation	Mouse model of cardiac hypertrophy	[35]
	miR‐29	COL1A1, COL3A1	Inhibits collagen synthesis, reducing EMT and cardiac fibrosis.	Mouse model of myocardial infarction	[36]
	miR‐133	RhoA, Cdc42	Inhibits EMT and reduces fibrosis and hypertrophy in the heart by targeting RhoA and Cdc42.	Mouse model of cardiac fibrosis	[37]
**Lung**	miR‐200 family	ZEB1, ZEB2	Inhibits EMT by downregulating ZEB1/2; preserves epithelial phenotype in lung tissue.	Mouse model of lung fibrosis	[38]
	miR‐21	PTEN, TGF‐β	Promotes EMT and pulmonary fibrosis by targeting PTEN and activating TGF‐β signaling.	Mouse model of idiopathic pulmonary fibrosis	[39]
	miR‐214	β‐catenin, EZH2	Enhances EMT, contributing to lung fibrosis by targeting β‐catenin and EZH2.	Mouse model of lung fibrosis	[40]
	miR‐29b	Collagen, TGF‐β	Inhibits collagen deposition and EMT in lung tissue, reducing fibrosis.	Mouse model of lung fibrosis	[41]
	miR‐155	SOCS1, SHIP1	Promotes EMT and enhances lung cancer progression by downregulating SOCS1 and SHIP1.	Mouse model of lung cancer	[42]
**Head and neck**	miR‐203	SLUG	Inhibits EMT and reduces metastasis in head and neck cancers by targeting SLUG.	Mouse model of head and neck squamous cell carcinoma	[43]
	miR‐205	ZEB1, ZEB2	Inhibits EMT by targeting ZEB1/2, suppressing invasion and metastasis.	Mouse model of head and neck cancer	[44]
	miR‐21	PTEN, PDCD4	Promotes EMT and invasiveness in head and neck cancers by targeting PTEN and PDCD4.	Mouse model of head and neck squamous cell carcinoma	[45]
	miR‐375	JAK2, PDK1	Inhibits EMT and tumor progression by targeting JAK2 and PDK1, reducing metastatic potential	Mouse model of oral squamous cell carcinoma	[46]
	miR‐34a	SNAIL1, SNAIL2	Inhibits EMT and enhances the sensitivity of head and neck cancer cells to treatment by targeting SNAIL transcription factors.	Mouse model of head and neck cancer	[47]
**Renal**	miR‐192	ZEB2, TGF‐βR1	Inhibits EMT and renal fibrosis by targeting ZEB2 and TGF‐β receptor 1	Mouse model of renal fibrosis	[48]
	miR‐21	PTEN, TGF‐β	Promotes EMT and fibrosis in the kidneys by inhibiting PTEN and activating TGF‐β signaling	Mouse model of renal fibrosis	[49]
	miR‐29	COL1A1, COL3A1	Inhibits collagen deposition and EMT, reducing renal fibrosis.	Rat model of renal fibrosis	[32]
	miR‐200b	ZEB1, ZEB2	Inhibits EMT by downregulating ZEB1/2, reducing fibrosis, and preserving kidney function	Mouse model of diabetic nephropathy	[50]
	miR‐146a	TRAF6, IRAK1	Inhibits EMT and inflammation, reducing renal fibrosis by targeting TRAF6 and IRAK1.	Mouse model of renal fibrosis	[51]
**Oral esophagus**	miR‐21	PTEN, PDCD4	Promotes EMT and enhances invasiveness in esophageal squamous cell carcinoma by targeting PTEN and PDCD4.	Mouse model of esophageal cancer	[52]
	miR‐31	SATB2, FZD3	Promotes EMT and invasion in oral squamous cell carcinoma by targeting SATB2 and FZD3	Mouse model of oral cancer	[53]
	miR‐375	JAK2, IGF1R	Inhibits EMT and tumor progression in oral esophageal cancer by targeting JAK2 and IGF1R	Mouse model of esophageal cancer	[54]
	miR‐200c	ZEB1, ZEB2	Inhibits EMT by targeting ZEB1/2, reducing metastasis and tumor invasiveness	Mouse model of esophageal cancer	[55]
	miR‐124	SLUG, TWIST1	Suppresses EMT and tumor progression by targeting SLUG and TWIST1 in oral squamous cell carcinoma	Mouse model of oral cancer	[56]
**Breast**	miR‐200	ZEB1, ZEB2	Inhibits EMT by downregulating ZEB1/2, reducing metastasis, and maintaining the epithelial phenotype	Mouse model of breast cancer	[57]
	miR‐21	PTEN, PDCD4	Promotes EMT and enhances breast cancer progression by targeting PTEN and PDCD4	Mouse model of breast cancer	[58]
	miR‐34a	SNAIL1, SNAIL2	Inhibits EMT and enhances sensitivity to chemotherapy by targeting SNAIL transcription factors	Mouse model of breast cancer	[59]
	miR‐145	SMAD3, TGF‐β	Inhibits EMT and reduces metastasis by targeting SMAD3 and TGF‐β signaling pathways	Mouse model of breast cancer	[60]
**Glioblastoma**	miR‐21	PDCD4, PTEN	Promotes EMT and enhances tumor invasiveness in glioblastoma by targeting PDCD4 and PTEN	Mouse model of glioblastoma	[61]
	miR‐125b	p53, MMP13	Inhibits EMT by targeting p53 and MMP13, reducing glioblastoma invasiveness	Mouse model of glioblastoma	[62]
	miR‐9	E‐cadherin, β‐catenin	Promotes EMT by downregulating	Mouse model	[63]

Abbreviations: IRAK1, interleukin‐1 receptor‐associated kinase 1; PDCD4, programmed cell death protein 4; PTEN, phosphatase and TENsin homolog; TGF‐β, transforming growth factor‐β; TIMP3, tissue inhibitor of metalloproteinase 3.

## 4 Role of Signaling Pathways Involved in EMT in Cancer Conditions

### 4.1 TGF‐β Pathway

**FIGURE 2:**
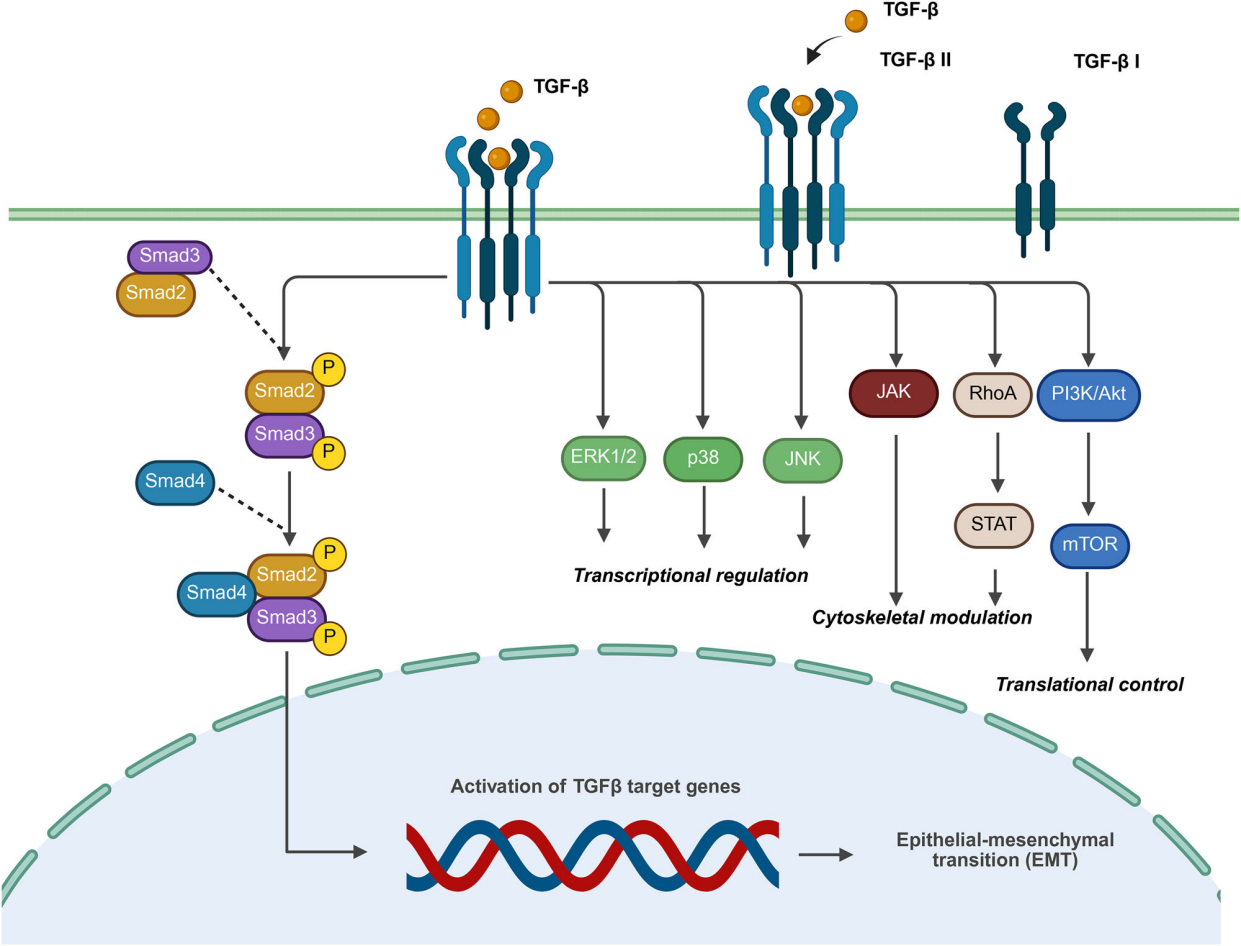
Graphical representation of TGF‐β signaling through canonical and noncanonical pathways involved in the important cellular activities of EMT. TGF‐β ligands bind to the TGF‐β receptor complex, comprising type II and type I receptors, which activate the intracellular signaling cascades. The canonical Smad‐dependent pathway begins with the phosphorylation of Smad2 and Smad3, leading to their association with Smad4. This complex then moves to the nucleus to regulate the transcription of genes associated with TGF‐β, including those that promote the EMT. TGF‐β also activates noncanonical pathways, including the MAPK pathways (ERK1/2, p38, and JNK), which regulate transcription, and the JAK/STAT and RhoA signaling pathways, which affect cytoskeletal dynamics and cellular motility. In addition, the PI3K/Akt/mTOR pathway influences cell survival and proliferation.

The TGF‐β pathway facilitates EMT, an important biological process. Exposure to ionizing radiation activates the TGF‐β pathway, leading to the induction of EMT in cancer cells via the phosphorylation of Smad2 and Smad3. Suppression of GF‐β signaling reduces radiation by inhibiting EMT and CSC programs [64]. Figure 2 illustrates TGF‐β ligand binding to the TGF‐β receptor complex, which consists of Types I and II receptors that activate intracellular signaling cascades. The homeobox family transcription factor distal homeobox 2 (DLX2) plays a role in radiation‐induced EMT. Furthermore, downregulation of DLX2 can reverse the effects of radiation‐induced EMT and CSC marker expression in lung cancer cells [65]. EMT cells are CSCs among breast cancer cells. These properties can contribute to radioresistance by increasing antioxidant defense and reducing intracellular ROS levels. The neurotrophin receptor‐interacting MAGE homolog (NRAGE) in the nucleoli of esophageal cancer cells has been linked to their resistance to radiation during TGF‐β and radiation‐induced EMT activation [66]. Berberine has the potential to improve radiosensitivity in nasopharyngeal carcinoma by inhibiting EMT and suppressing TGF‐β1, which downregulates the expression of specific protein 1 (Sp1) [67].

### 4.2 Notch Pathway

**FIGURE 3:**
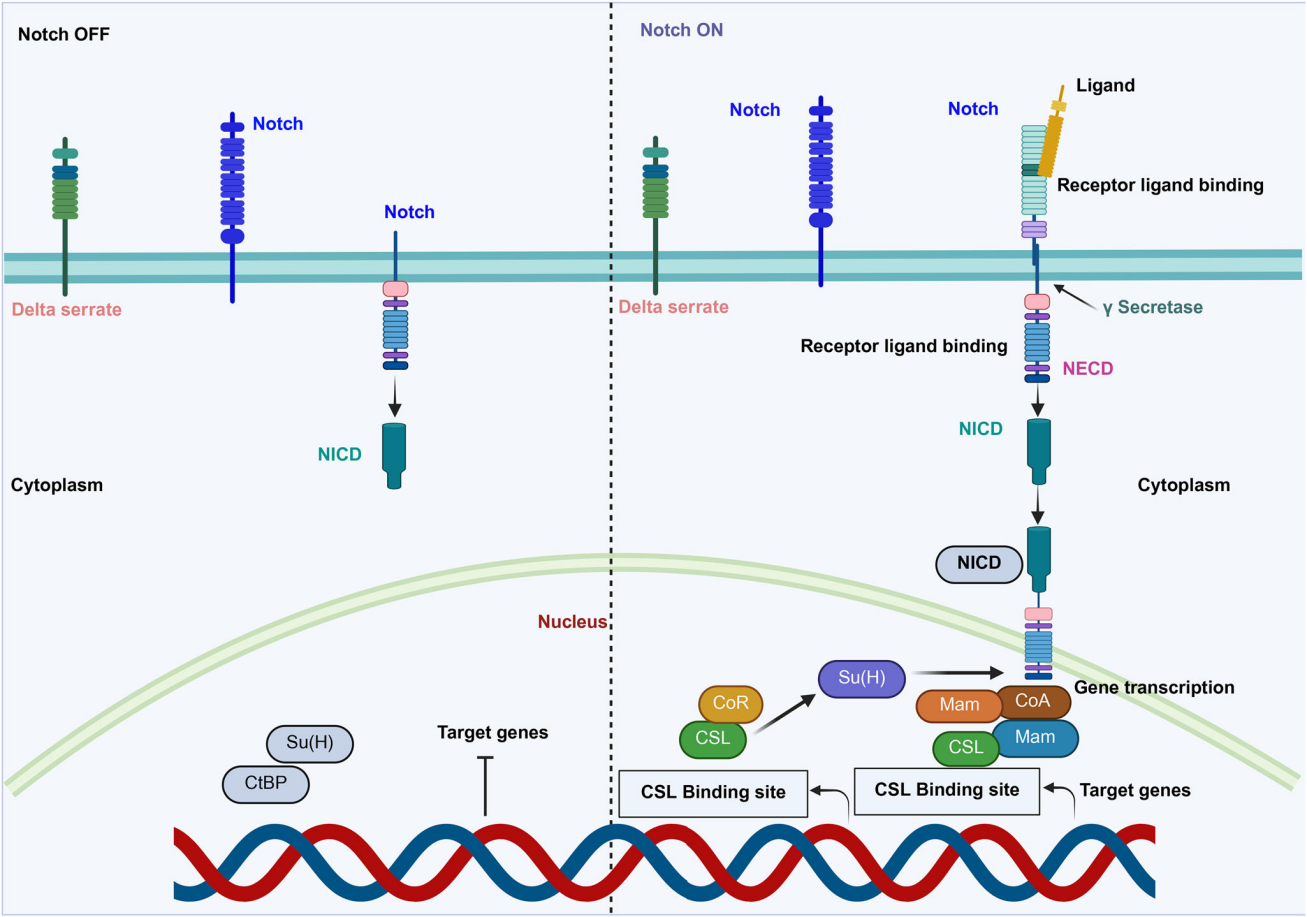
The graphic illustrates the Notch signaling pathway in its “OFF” and “ON” states. In the “OFF” state, Delta Serrate ligands do not bind to the Notch receptor, keeping it inactive, and the intracellular domain (NICD) attaches to the cell membrane. In the “ON” state, ligand binding activates the receptor, leading to its cleavage by γ‐secretase, which releases NICD. The NICD then enters the nucleus, where it converts the transcription factor CSL from a repressor to an activator. Together with the coactivator Mam, this complex initiates the transcription of target genes that are crucial for cellular functions.

The Notch signaling pathway includes Notch 1–4 and ligands DLL 1/3/4 and Jagd 1/2. In gastric cancer, tangerine has been shown to inhibit Notch‐1, improve radiosensitivity, and prevent radiation‐induced EMT in laboratory and animal models. Rhamnetin inhibits the Notch‐1 pathway in non‐small cell lung cancer (NSCLC), reducing radioresistance and altering EMT characteristics [68]. In addition, Zhou et al. demonstrated that inhibiting Notch signaling using γ‐secretase inhibitors or Notch1/2‐siRNA can improve the radiosensitivity of glioma stem cells and nasopharyngeal carcinoma (NBC) cells [26]. In breast cancer, EMT is initiated by the ionizing radiation‐induced activation of NF‐κB. Radiation exposure elevates NF‐κB levels, which drives EMT transcription factors such as Twist and Snail [69]. NF‐κB promotes EMT, radioresistance, JAK/STAT3 activation, and IL‐6 production in breast CSCs. Radiation activates NF‐κB, thereby increasing the expression of mesenchymal markers and the invasiveness of lung cancer cells. Figure 3 shows how Delta and Serrate ligands bind to the Notch receptor, activating the Notch signaling pathway and releasing the Notch intracellular domain (NICD) via γ‐secretase. NICD enters the nucleus and interacts with CSL and Mam to initiate transcription of target genes. In gastric cancer, radiation therapy activates NF‐κB, which induces EMT and enhances the migration and invasion of gastric cancer cells by overexpressing EMT‐related genes [70]. In glioblastomas, radiation activates the NF‐κB pathway, which is crucial for maintaining the stem‐like characteristics of cancer cells. NF‐κB enhances DNA repair mechanisms and EMT marker expression, thereby contributing to radioresistance. Suppressing NF‐κB signaling may enhance radiation sensitivity in glioblastoma cells, and research suggests that the NF‐κB pathway may affect radioresistance [71]. Radiation exposure activates NF‐κB signaling in esophageal cancer, promoting EMT and cell invasiveness via pro‐inflammatory cytokines. Targeting NF‐κB could help to treat radioresistance in this cancer [72].

### 4.3 WNT/β‐Catenin Pathway

**FIGURE 4:**
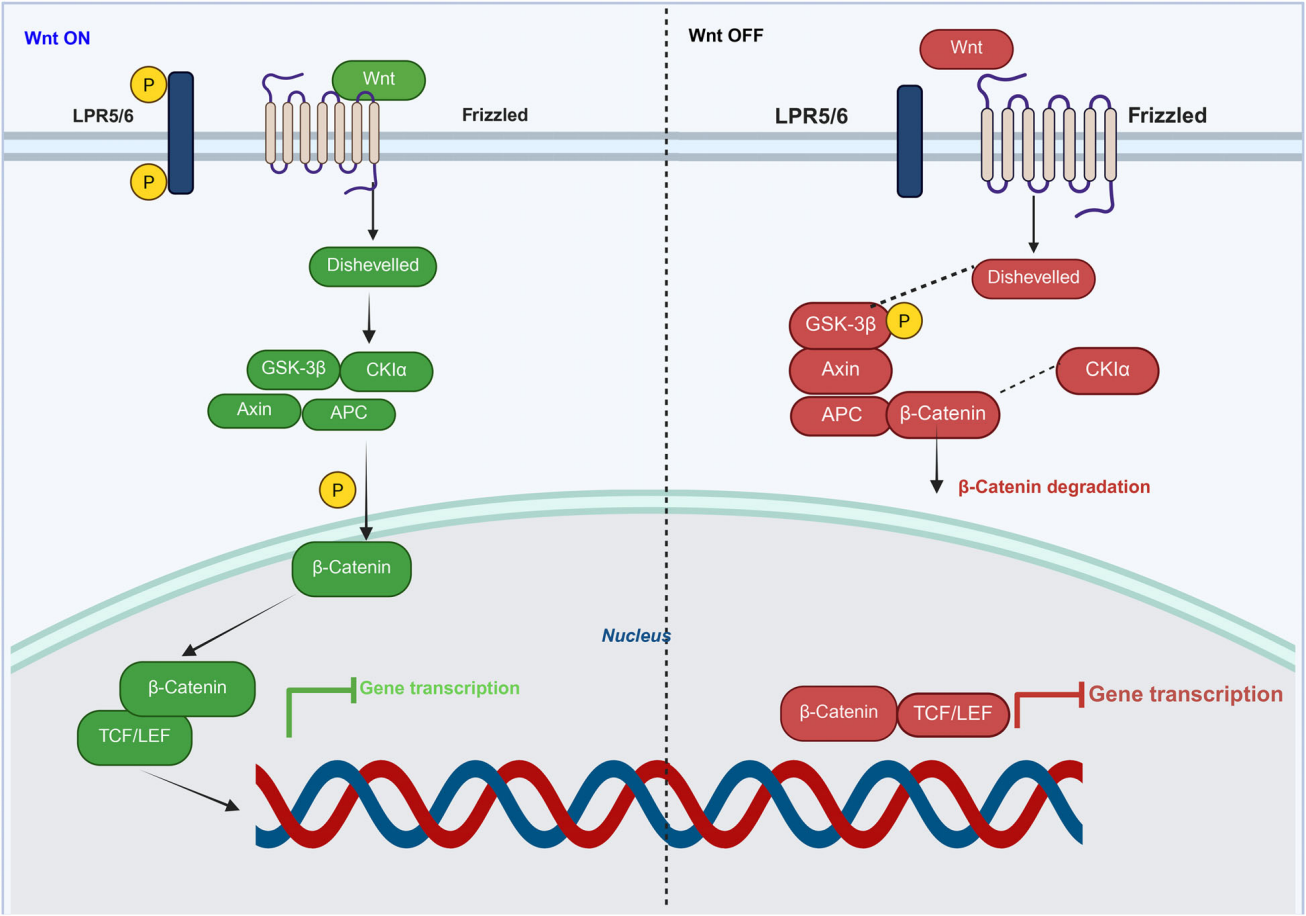
Illustration of the Wnt signaling system in the “OFF” and “ON” states. In the “OFF” mode, frizzled (Fz) and LRP5/6 receptors are inactive. In the absence of Wnt, the destruction complex phosphorylates β‐catenin, inactivates Dishevelled (Dvl), and prevents gene transcription by marking β‐catenin for degradation. In the “ON” state, Wnt binds to Frizzled and LRP5/6, activating dishevelled and preventing β‐catenin degradation. Stabilized β‐catenin enters the nucleus to stimulate target gene transcription by interacting with TCF/LEF transcription factors, thereby driving various biological processes.

Radiation‐induced EMT is enhanced by abnormal Wnt/β‐catenin signaling. Radiation‐activated Wnt ligands interact with LRP 5/6 co‐receptors and serpentine receptors. This combination phosphorylates the Dishevelled (Dsh) protein, preventing β‐catenin degradation by GSK3β [73]. Subsequently, β‐catenin joins TCF and LEF transcription factors to activate Wnt target genes in the nucleus. Esophageal Squamous Cell Carcinoma (ESCC) cells that resist RT develop Wnt signaling and EMT. Blocking the Wnt/β‐catenin pathway in esophageal cancer cells may enhance radiosensitivity and reverse EMT [74]. Radioresistance in neural progenitor cells (NPC) is induced by the activation of the Wnt/β‐catenin signaling pathway, which leads to EMT phenotypes by suppressing FOXO3a. Bastos et al. found that radiation‐exposed colorectal cancer cells exhibit an EMT phenotype, activating Wnt/β‐catenin‐dependent TCF/LEF and conferring radioresistance [75]. Cojoc et al. found that the Wnt/β‐catenin pathway regulates ALDH activity, promoting an EMT phenotype that enhances radioresistance in prostate cancer progenitor cells. Conversely, inhibition of this pathway reduces ALDH activity and sensitizes prostate cancer cells to RT. In gastric cancer, radiation triggers Wnt pathway activation, leading to EMT and increased resistance to RT [76]. Zhao et al. reported the mechanisms by which Wnt signaling regulates radioresistance in glioblastoma by repairing DNA damage. Wnt signaling helps glioblastoma cells survive radiation by repairing DNA damage. This mechanism helps glioma cells maintain their stemness, making them aggressive and resistant to treatment [77]. Wnt signaling also plays a role in radioresistance of head and neck squamous cell cancer (HNSCC). This increase is associated with enhanced DNA repair capability and stemness [78]. Figure 4 shows that Wnt co‐receptors are LRP5 and LRP6. Deactivation of β‐catenin allows its disintegration owing to its inactive status. In the ON state, Wnt phosphorylation activates dishevels and stabilizes β‐catenin, thereby facilitating gene transcription.

### 4.4 ERK Pathway

**FIGURE 5:**
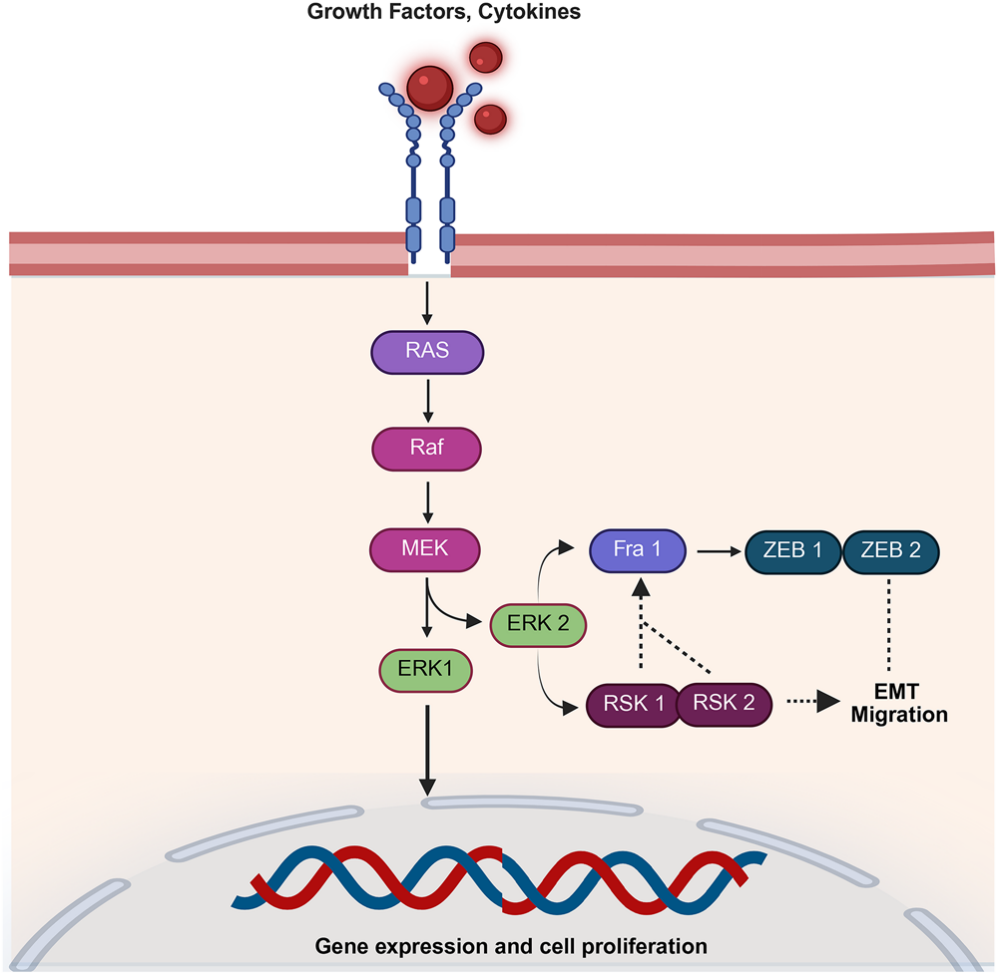
The graphic illustrates how cytokines and growth factors regulate gene expression, cell division, and epithelial–mesenchymal transition (EMT) migration through the signaling networks. They activate Raf and RAS by binding to membrane receptors, leading to MEK phosphorylation and ERK protein activation. These pathways influence EMT and migration by regulating Fra1, which in turn controls the expression of ZEB1 and ZEB2. ERK also activates RSK1 and RSK2, impacting EMT factors and promoting invasion, migration, increased cell proliferation, and gene expression. This pathway highlights the role of EMT in cancer physiology and its pathology.

ERK1 and ERK2 signaling through the MAPK pathway regulates cell survival, differentiation, and proliferation during stress. Enhanced ERK signaling may lead to resistance to radiation and chemotherapy [79]. Blocking ERK1/2 and Akt in prostate cancer cell lines increases radiosensitization. AKT and ERK1/2 phosphorylation and caspase‐3 inhibition may render NSCLC cells resistant to radiation treatment [80]. Therefore, plasminogen activator inhibitor‐1 may affect resistance. The standard form of CD44 activates ERK, thereby preventing radiation‐induced EMT in pancreatic cancer cells [81]. NSCLC cells undergo EMT after radiation exposure, which results in increased ERK phosphorylation. This results in increased levels of mesenchymal markers, such as vimentin and N‐cadherin, and decreased levels of the epithelial marker E‐cadherin. The expression of the EMT transcription factors Snail and Twist was also increased. Research suggests that ERK pathway inhibitors, including SCH772984, may reverse EMT and enhance cancer cell sensitivity to radiation [82]. In breast cancer, radiation activates ERK, leading to GSK‐3β phosphorylation, which stabilizes Snail and promotes EMT, making cells more aggressive and radiation‐resistant [83]. Pathway‐specific inhibitors may block EMT and enhance the radiation sensitivity of breast cancer cells. Research has linked radiation‐induced EMT in esophageal cancer to the ERK pathway, which regulates gene expression and contributes to radioresistance. Targeting the ERK pathway and reversing EMT may improve the effectiveness of radiation therapy in glioblastoma and HNSCC [84]. Cytokines and growth factors influence EMT and cell migration through signaling networks. Raf activates MEK, which in turn activates ERK and regulates EMT transcription factors such as Fra1, ZEB1, and ZEB2. This process promotes cell invasion and proliferation, highlighting the role of EMT in cancer progression (Figure 5).

## 5 Tumor Microenvironment and Radiation‐Induced EMT Implications in Cancer Progression

Radiation therapy, a common treatment for various malignancies, paradoxically promotes EMT and enhances tumor growth, metastasis, and resistance to treatment. Radiation increases TGF‐β, activating SMAD2/3 and the EMT‐related genes Twist and Snail, while noncanonical TGF‐β signaling and NF‐κB pathway activation also contribute to EMT. The upregulation of Wnt/β‐catenin and Notch signaling pathways further enhances EMT and cell survival. Radiation‐induced EMT enriches CSCs and promotes a pro‐inflammatory and immunosuppressive tumor microenvironment. Epigenetic alterations, such as the downregulation of miR‐200c and upregulation of miR‐21, also occur [85]. The TGF‐β pathway, an essential regulator of EMT, is inadvertently activated in cancer cells due to radiation‐induced DNA damage and oxidative stress, thereby amplifying cellular stress responses. The binding of TGF‐β to receptors on cancer cells initiates a cascade of events that activate SMAD transcription factors, which are translocated to the nucleus and initiate gene expression, thereby promoting EMT. Various non‐SMAD pathways, including the PI3K/AKT and MAPK pathways, modulate cell motility and survival [86]. Radiation induces ROS production, which leads to oxidative damage in cells. Cancer cells exhibit elevated production of antioxidants, including glutathione peroxidase (GPx) and superoxide dismutase (SOD), which counteract the effects of ROS. ROS play a crucial role in regulating EMT in cancer, amplifying TGF‐β signaling, promoting EMT via SMAD‐dependent and SMAD‐independent routes, and activating the NF‐κB pathway, thereby contributing to EMT and immune evasion [87]. ROS‐mediated EMT is important, including resistance to radiation and chemotherapy, promotion of survival pathways, enrichment of CSCs, and EMT, ultimately leading to metastasis and recurrence. Targeting ROS‐mediated EMT offers promising therapeutic strategies for cancer treatment, with antioxidants such as *N*‐acetylcysteine and tempol to reduce oxidative stress and inhibit EMT, and NOX inhibitors such as GKT137831 to block ROS production [88]. TGF‐β inhibitors, such as galunisertib and LY2157299, can prevent EMT, while NF‐κB inhibitors, such as bortezomib and curcumin, can reduce inflammation. Redox‐sensitive drug delivery systems have also been developed to selectively release EMT inhibitors under oxidative tumor microenvironment conditions [89]. By targeting ROS‐mediated EMT, novel therapeutic approaches can be developed to improve cancer treatment outcomes and overcome resistance. Combining targeted therapies with radiation or chemotherapy may enhance the treatment efficacy and reduce recurrence. Understanding the complex interactions between radiation, EMT, and the tumor microenvironment is crucial for developing effective therapeutic strategies. Overall, targeting ROS‐mediated EMT holds promise for improving cancer therapy and patient outcomes. Further research is required to explore the therapeutic potential of targeting EMT in cancer treatment.

## 6 EMT as a Therapeutic Target Approach to Radioresistance in Clinical Trials

EMT has a significant effect on the treatment of radiation‐induced tissue damage, especially during RT. It is important to investigate the EMT pathway as a possible treatment method because it plays a major role in the resistance of cancer cells to radiation [90]. This approach to EMT is a treatment area of interest, including ongoing and future clinical studies and drugs designed to reduce radiation‐induced EMT. Table 2 shows the new and ongoing clinical studies on targeted EMT in various types of cancers and related diseases linked to them.

**TABLE 2:** Recent clinical trial data on EMT have targeted various cancer conditions for therapeutic approaches.

NCT number	Study title	Study status	Conditions	Interventions	Biomarkers	Sex	Age	Phases
NCT02602938	Aspirin on CTCs of advanced breast and colorectal cancer	UNKNOWN	EMT|circulating tumor cells	DRUG: Aspirin	PIK3CA ↑, COX‐2 ↑.	ALL	Adult, Older_ Adult	PHASE2
NCT03509779	Prognostic and predictive value of EMT in localized lung cancer	RECRUITING	NSCLC, Stages I, II, IIIA, IIIB|surgery|progression|EMT		Snail ↑, TWIST1 ↑, slug, and FOXC2 ↑.	ALL	Adult, Older_ Adult	–
NCT04021394	Influence of EMT on CTCs and disease progression in prostate cancer	COMPLETED	Prostate cancer	DIAGNOSTIC_TEST: CellSearch CTC platform|DIAGNOSTIC_TEST: Parsortix CTC platform	E‐Cadherin ↑, N‐Cadherin ↑, Zeb‐1 ↑, Vimentin ↑, and Twist ↑.	MALE	Adult, Older_ Adult	–
NCT05550415	The role of simvastatin in the EMT process of breast cancer	RECRUITING	Triple negative breast cancer|chemotherapy effect|simvastatin adverse reaction	DRUG: Simvastatin 40 mg|DRUG: Placebo	Vimentin ↓.	FEMALE	Adult, Older_ Adult	PHASE2
NCT02221882	A study of LY3164530 in participants with cancer	COMPLETED	Neoplasms|neoplasm metastasis	DRUG: LY3164530	MET ↑, EGFR ↑, KRAS ↑.	ALL	Adult, Older_ Adult	PHASE1
NCT02579460	Reflux‐induced oxidative stress in Barrett's esophagus: response, repair, and epithelial–mesenchymal‐transition	COMPLETED	Barrett's esophagus|gastroesophageal reflux disease	OTHER: cessation of acid suppressing medications	HIF‐1 alpha ↑, VEGF ↑, ZEB 1 ↑, and Vimentin ↑, ↓.	ALL	Adult, Older_ Adult	NA
NCT04137523	Investigate eNAMPT in multiple myeloma biology and establish its role in disease progression	UNKNOWN	Multiple myeloma	–	eNAMPT ↑, IL‐6 ↑, β2‐microglobulin ↑.	ALL	Adult, Older_ Adult	
NCT05541822	To evaluate the efficacy, safety, tolerability, and pharmacokinetic profile of ABN401 in patients with advanced solid tumors harboring c‐MET dysregulation	RECRUITING	Advanced solid tumors	DRUG: ABN401	MET exon 14 skipping ↑, c‐MET protein overexpression (IHC) ↑, phosphor‐MET ↑	ALL	Adult, Older_ Adult	PHASE2
NCT03138083	OMO‐1 in Solid Malignancies	TERMINATED	Neoplasms	DRUG: OMO‐1	MET exon 14 skipping ↑, p‐MET ↓,	ALL	Adult, Older_ Adult	PHASE1|PHASE2
NCT01697072	First‐line treatment for locally advanced or metastatic MET‐positive gastric, lower esophageal, or GEJ adenocarcinoma	TERMINATED	Gastric cancer	DRUG: Rilotumumab|OTHER: Placebo|DRUG: Epirubicin|DRUG: Cisplatin|DRUG: Capecitabine	MET ↑, HGF ↓.	ALL	Adult, Older_ Adult	PHASE3
NCT03381326	CTC, free DNA, stem cells, and EMT‐related antigens as biomarkers of activity of cabazitaxel in CRPC.	ACTIVE_NOT_RECRUITING	Prostate cancer|metastatic cancer|castration‐resistant prostate cancer|circulating tumor cells	PROCEDURE: blood and FFPE sample collection	AR‐V7 ↑, AR ↑, EPCAM ↑, PSMA ↑, MDK ↑, HPRT1 ↑, AKR1C3 ↑, and ALDH 1 ↑	MALE	Adult, Older_ Adult	
NCT06055660	Moesin expression in clear cell renal cell carcinoma	COMPLETED	Renal cell carcinoma	GENETIC: Immunohistochemical detection of Moesin in Clear cell Renal Cell Carcinoma	—	ALL	Adult, Older_, Adult	–
NCT03030417	Indenoisoquinoline LMP744 in adults with relapsed solid tumors and lymphomas	COMPLETED	Solid tumors|lymphoma	DRUG: LMP744	SLFN11 ↑, RAD51 ↑, pKAP1 ↑, γH2AX ↑, and Cleaved caspase‐3 ↑.	ALL	Adult, Older_ Adult	PHASE1
NCT02940977	Establishment and clinical assessment of a prostate cancer risk model based on the updated CTC detection technique	UNKNOWN	Prostatic neoplasms|prostatic adenoma	OTHER: Blood draws	VIM ↑, Twist, SNAL 1 ↑, N‐cadherin ↑, EPCAM ↓, and CK8/18/19 ↓.	MALE	Adult, Older_ Adult	
NCT02412462	Phase I Dose escalation study of AB‐16B5 in subjects with an advanced solid malignancy	COMPLETED	Solid tumor|metastatic cancer	DRUG: AB‐16B5	EPCAM ↓, CK8/18/19 ↓, VIM ↑, Twist ↑, SNAL 1 ↑, N‐cadherin ↑, ALDH 1 ↑, and CD44 ↑.	ALL	Adult, Older_ Adult	PHASE1
NCT04323917	Detection of high expression levels of EMT‐transcription factor mrnas in patients with pancreatic cancer and their diagnostic potential	UNKNOWN	Pancreatic cancer	DIAGNOSTIC_TEST: Liquid biopsy	TWIST1 ↑, ZEB2 ↑, CDH1 (E‑cadherin) ↓.	ALL	Adult, Older_ Adult	
NCT01927354	Study on the interplay between Twist1 and other EMT regulators through microRNA‐29 family.	UNKNOWN	Head‐and‐neck squamous cell carcinoma		TWIST1 ↑, miR‐29a/b/c ↑, SIN3A ↓, Snail ↑ ↓.	ALL	Adult, Older_ Adult	
NCT05176665	EMB‐01 in patients with advanced/metastatic gastrointestinal cancers	RECRUITING	Neoplasms|neoplasm metastasis|metastatic gastrointestinal carcinoid tumor	DRUG: EMB‐01	c‐MET ↑, EGFR ↑, p‐MET ↓, p‐EGFR ↓.	ALL	Adult, Older_ Adult	PHASE1|PHASE2
NCT04817501	Phenotypic spectrum of CTCs in tumors of the female reproductive system	COMPLETED	Breast cancer|ovarian cancer|endometrial cancer	OTHER: Taking 5 mL of venous blood at different time intervals|OTHER: Taking 5 mL of EDTA‐stabilized ascitic fluid sampled during laparoscopy if any	EpCAM ↑, CKs ↑, Ki‐67 ↑, HER 2↑.	FEMALE	Adult, Older_ Adult	
NCT05940116	A Phase I clinical study of HS‐20117 in participants with advanced solid tumors	NOT_YET_RECRUITING	Non‐small cell lung cancer|solid tumor	DRUG: HS‐20117	EGFR mutation ↑, p‐EGFR ↓, p‐MET ↓,	ALL	Adult, Older_ Adult	PHASE1
NCT06147037	A Phase 1, dose‐escalation study of [225Ac]‐FPI‐2068 in adult patients with advanced solid tumors	RECRUITING	Advanced solid tumor|metastatic colorectal carcinoma|head and neck squamous cell carcinoma|non‐small cell lung cancer|pancreatic ductal adenocarcinoma	DRUG: FPI‐2053|DRUG: [111In]‐FPI‐2107|DRUG: [225Ac]‐FPI‐2068	—	ALL	Adult, Older_ Adult	PHASE1
NCT06417008	A study of HS‐20117 combined with aumolertinib in participants with advanced non‐squamous non‐small cell lung cancer	NOT_YET_RECRUITING	Non‐squamous non‐small cell lung cancer	DRUG: HS‐20117|DRUG: Aumolertinib	EGFR ↑, MET ↑, p‐EGFR ↓, p‐MET ↓.	ALL	Adult, Older_ Adult	PHASE2|PHASE3
NCT05110196	Study of capmatinib in indian patients with MET Exon 14 skipping mutation positive advanced NSCLC.	RECRUITING	Non‐small cell lung carcinoma	DRUG: Capmatinib 150 mg|DRUG: Capmatinib 200 mg	MET exon 14 skipping mutation ↑, MET gene amplification ↑, p‐MET ↓, p‐AKT/ERK ↓.	ALL	Adult, Older_ Adult	PHASE4
NCT01441128	−02341066 and PF‐00299804 for advanced non‐small cell lung cancer	TERMINATED	Carcinoma, non‐small cell lung|adenocarcinoma|carcinoma, squamous cell|carcinoma, large cell	DRUG: PF‐02341066/PF‐00299804|DRUG: PF‐02341066/PF‐00299804	Phospho–MET ↓, phosphor–EGFR ↓, HGF/sMET →, (KRAS, EGFR‐T790M, MET) →.	ALL	Adult, Older_ Adult	PHASE1
NCT02609776	Study of amivantamab, a human bispecific EGFR, and cMet antibody in participants with advanced non‐small cell lung cancer	ACTIVE_NOT_RECRUITING	Non‐small‐cell lung cancer	DRUG: Amivantamab|DRUG: Amivantamab|DRUG: Lazertinib|DRUG: Carboplatin|DRUG: Pemetrexed	MET ↑, EGFR ↑.	ALL	Adult, Older_ Adult	PHASE1

*Note*: Clinical trial data were sourced from the official registry, ClinicalTrials.gov (https://clinicaltrials.gov/).

Abbreviations: C‐MET, cellular mesenchymal epithelial transition factor; CRPC, castration‐resistant prostate cancer; CTC, circulating tumor cells; eNAMPT, extracellular nicotinamide phosphoribosyl transferase; GEJ, gastroesophageal junction; NSCLC, non‐small cell lung cancer.

Natural drugs that modulate the immune system are used in cancer treatment. Current research aims to use natural chemicals to target the EMT process more safely, possibly in combination with other therapies. Effective natural medicines for lowering EMT markers include curcumin, lycopene, silymarin, and sulforaphane [91]. The drugs listed in Table 3 inhibit EMT by targeting specific signaling pathways modulated by cancer cells. During radiation treatment, natural medicines interrupt EMT signs and affect pathways such as EGFR, TGF‐β, Notch, Wnt, ERK, mTOR, and NF‐κB, which are associated with increased radioresistance in cancer.

**TABLE 3:** Summary of drugs that inhibit the effects of EMT during radiation therapy for cancer treatment.

Cancer types	Drug	Target mechanism	Toxicity	EMT	Ref
**Liver cancer**	Sorafenib	VEGFR, RAF kinases	Hepatotoxicity, hand–foot syndrome	Inhibits EMT by blocking TGF‐β signaling, but resistance can induce EMT in residual cells	[92]
	Doxorubicin	DNA intercalation, Topoisomerase II	Cardiotoxicity, hepatotoxicity	Induces EMT through activation of TGF‐β and NF‐κB pathways, promoting liver cancer progression	[93]
	Cisplatin	DNA crosslinking	Nephrotoxicity, neurotoxicity	induce EMT in liver cancer cells, leading to increased invasion and metastasis	[94]
**Breast Cancer**	Trastuzumab	HER2	Cardiotoxicity, infusion reactions	Inhibits EMT by targeting HER2, but resistance may activate EMT pathways	[95]
	Paclitaxel	Microtubule stabilization	Peripheral neuropathy, myelosuppression	Promotes EMT by increasing TGF‐β signaling, leading to enhanced metastasis	[96]
	Doxorubicin	DNA intercalation, Topoisomerase II	Cardiotoxicity, myelosuppression	Induces EMT through ROS production, promoting tumor invasion	[97]
**Lung cancer**	Erlotinib	EGFR tyrosine kinase inhibitor	Rash, diarrhea, hepatotoxicity	Inhibits EMT by targeting EGFR, but resistance may activate alternative EMT pathways	[98]
	Cisplatin	DNA crosslinking	Nephrotoxicity, ototoxicity, neurotoxicity	Induces EMT through activation of TGF‐β and Wnt/β‐catenin signaling, promoting lung cancer metastasis	[99]
	Nivolumab	PD‐1 inhibitor	Immune‐related adverse events	Modulates EMT by altering the tumor microenvironment, potentially reversing EMT	[100]
**Head and neck**	Cetuximab	EGFR inhibitor	Skin rash, hypomagnesemia	Inhibits EMT by targeting EGFR, but resistance can lead to EMT activation and increased invasion.	[101]
	Cisplatin	DNA crosslinking	Nephrotoxicity, ototoxicity, neurotoxicity	Induces EMT, leading to increased invasiveness and metastasis in head and neck cancer cells	[102]
	Docetaxel	Microtubule stabilization	Myelosuppression, neuropathy	EMT by enhancing TGF‐β signaling, contributing to metastasis and resistance.	[96]
**Renal cancer**	Sunitinib	VEGFR, PDGFR, KIT inhibitor	Hypertension, cardiotoxicity, hand–foot syndrome	Inhibits EMT, but long‐term treatment can lead to resistance through EMT induction	[103]
	Sorafenib	VEGFR, RAF kinases	Hepatotoxicity, hand–foot syndrome	Inhibits EMT initially, but resistance can activate EMT pathways and increase metastasis	[92]
	Temsirolimus	mTOR inhibitor	Hyperglycemia, pneumonitis	induce EMT through mTOR signaling, contributing to tumor progression and drug resistance	[104]
**Oral esophageal cancer**	5‐Fluorouracil (5‐FU)	Thymidylate synthase inhibitor	Myelosuppression, mucositis	Promotes EMT by activating TGF‐β and Wnt/β‐catenin pathways, leading to increased metastasis	[105]
	Cisplatin	DNA crosslinking	Nephrotoxicity, neurotoxicity, ototoxicity	Induces EMT in esophageal cancer cells, contributing to resistance and metastasis	[106]
	Paclitaxel	Microtubule stabilization	Peripheral neuropathy, myelosuppression	metastasis in oral esophageal cancer	[107]
**Glioblastoma**	Temozolomide	DNA alkylation	Myelosuppression, hepatotoxicity	May induce EMT by activating TGF‐β and NF‐κB pathways, contributing to glioblastoma invasiveness	[108]
	Bevacizumab	VEGF inhibitor	Hypertension, thromboembolic events	Inhibits angiogenesis and EMT, but resistance can lead to enhanced EMT and tumor recurrence.	[109]
	Lomustine	DNA alkylation	Myelosuppression, pulmonary toxicity	induce EMT, leading to increased glioblastoma cell invasion and resistance	[110]

Abbreviations: NF‐kB, nuclear factor kappa B; PDGFR, platelet‐derived growth factor receptor; VEGF, vascular endothelial growth factor; VEGFR, vascular endothelial growth factor receptor.

**FIGURE 6:**
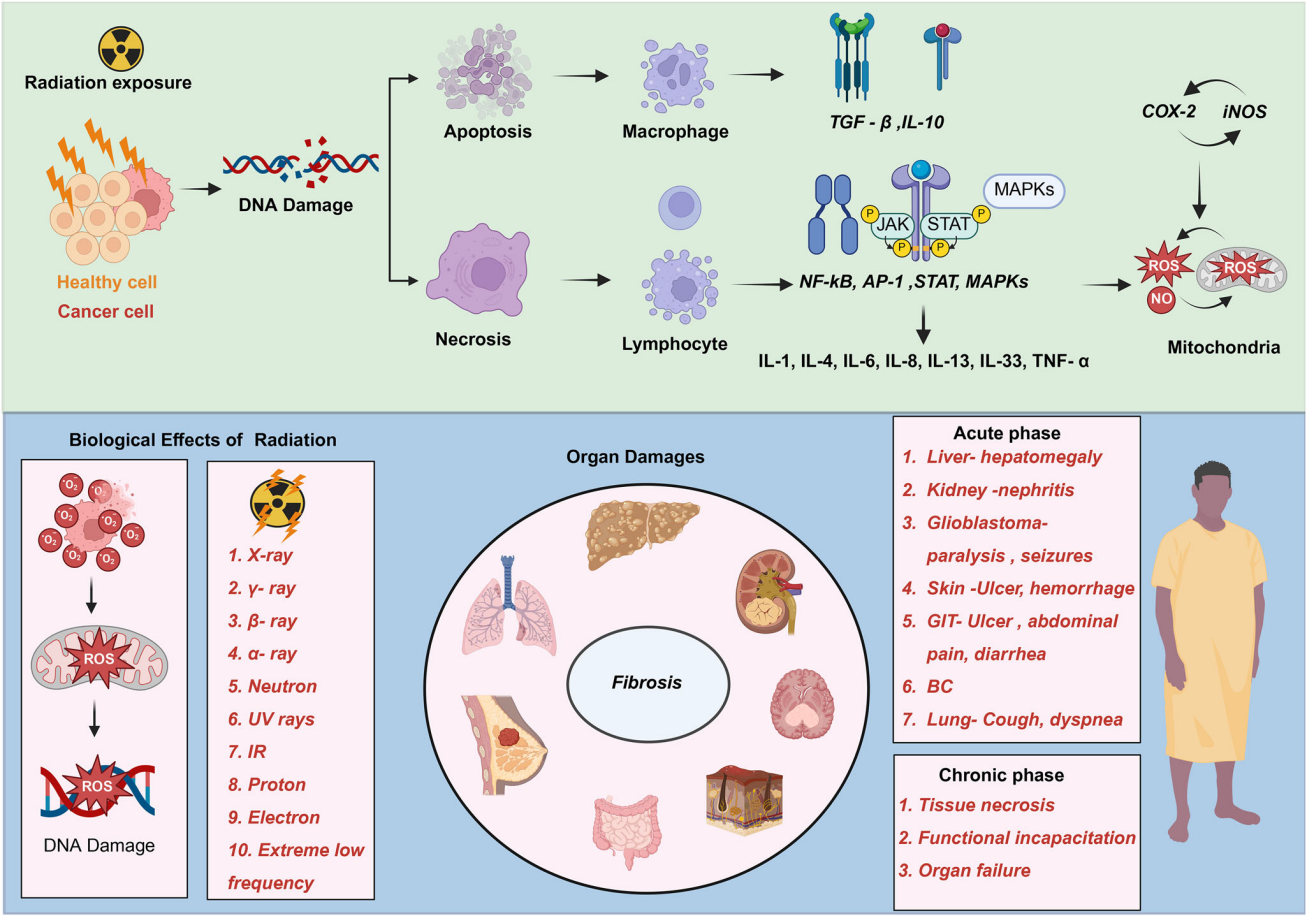
Radiation exposure triggers cellular reactions and organ damage, leading to radiation‐induced injuries. It damages DNA, causing both normal and cancerous cells to undergo apoptosis and necrosis, respectively. Immune cells, particularly macrophages and lymphocytes, respond by releasing anti‐inflammatory cytokines, such as TGF‐β and IL‐10, and pro‐inflammatory mediators, such as IL‐1 and TNF. These cytokines activate pathways that enhance inflammation. Mitochondria generate reactive oxygen species (ROS) and nitric oxide (NO), which contribute to oxidative stress and tissue injury. Various types of radiation, including x‐rays, gamma rays, and ultraviolet rays, can cause this damage. Following exposure, fibrosis often affects the liver, lungs, kidneys, skin, and brain. Acute symptoms may include hepatomegaly, renal nephritis, brain gliomas, and gastrointestinal issues, such as ulcers and diarrhea. Chronic exposure can result in tissue necrosis, cellular apoptosis, functional impairment, and organ failure, emphasizing the need for effective mitigation strategies.

Current therapeutic approaches focus on ongoing clinical trials of TGF‐β, Wnt, Notch, and ERK inhibitors. Studies have examined their effectiveness in reducing radiation‐induced fibrosis and EMT, potentially enhancing radiation therapy by targeting the associated signaling pathways [111]. As shown in Table 2, clinical trial data and ongoing research studies are currently evaluating compounds that inhibit this pathway in humans. Combination therapy uses radiation therapy along with tailored medications that specifically target the mechanisms involved in ECM production. Figure 6 shows that radiation exposure damages DNA, causes apoptosis and necrosis, and induces oxidative stress through ROS and nitric oxide. This results in tissue injury and inflammation, with acute symptoms such as organ inflammation and chronic effects such as fibrosis and organ failure.

This treatment may be more effective in delaying the onset of radioresistance when chemotherapeutic agents are combined with TGF‐β or Notch inhibitors. The immune system can be negatively affected by EMT; however, immunotherapy and targeted RT may mitigate this effect on the immune system. Emphasizing EMT in cancer treatment can enhance the effectiveness of radiation therapy and provide lasting benefits to patients [112]. Therefore, strategies to combat radiation resistance and prevent radiation‐induced EMT are essential. Medications targeting pathways such as TGF‐β, Notch, Wnt, and ERK are currently in clinical trials, offering hope for improved treatments against therapy resistance. Further research on ECM and EMT is required to understand their effects on radiation‐induced tissue damage. This will help to create therapies that maximize the benefits of radiation while protecting healthy tissues.

## 7 Current and Emerging Therapeutic Strategies for EMT in Radiation and Cancer Conditions

### 7.1 Multi‐Omics Approaches

Multi‐omics methodologies synthesize data at diverse molecular levels, including epigenomic, proteomic, metabolomic, and transcriptomic. Modern technology and software facilitate the precise characterization of EMT and the discovery of new therapeutic targets [113]. Genomic and transcriptomic analyses have clarified the changes in gene expression during EMT. Radiation responses and cancer metastasis influence EMT through the TGF‐β, Wnt/β‐catenin, and Notch signaling pathways, involving transcription factors such as Snail, Slug, ZEB1/2, and Twist [10]. Targeting these pathways enables RNA‐based therapies, such as antisense oligonucleotides and small molecules, to inhibit EMT. Monoclonal antibodies and proteolysis‐targeting chimeras (PROTACs) are two therapeutic agents under development that selectively target EMT proteins [114]. Metabolic resetting, which includes changes in glycolysis, lipid metabolism, and amino acid synthesis, occurs simultaneously with EMT. Metabolic inhibitors targeting fatty acid synthase or glycolytic enzymes may reduce the ability of cancer cells to endure radiation and EMT [115]. Single‐cell omics technology allows for detailed analysis of EMT at the cellular level, identifying specific cell subpopulations in tumors. This will aid in the development of targeted therapies [116]. The combination of multi‐omics data with systems biology enhances our understanding of the mechanisms underlying EMT. Artificial intelligence (AI) and machine learning are crucial for managing complex data and improving combination therapies that target multiple EMT pathways [117].

### 7.2 Gene Editing

Gene‐editing techniques in cancer and radiation therapy aim to inhibit or reverse drug resistance and the EMT by altering the genomic material. Deletion or modification of genes associated with EMT, such as Snail, Twist, and ZEB1, can reverse the mesenchymal phenotype of cancer cells, making them more responsive to treatment [118]. An enhanced immune response against tumors is a potential benefit of CRISPR technology, which modifies immune cells to more effectively recognize and target cancer cells undergoing EMT. CRISPR can help restore normal cellular functions and reduce the spread of cancer by reinstating or activating tumor suppressor genes. Gene editing targeting the CDH1 promoter may promote EMT reversal and help restore epithelial characteristics [119]. Gene editing affects epigenetic modifications by altering the markers that regulate the expression of genes associated with EMT. The CRISPR‐Cas9 system aids in the epithelial transition of cancer cells by modifying histone deacetylases and DNA methyltransferases [120]. The combination of gene editing with radiation and chemotherapy has the potential to improve the therapeutic efficacy. Owing to its significant role in EMT, inhibiting TGF‐β through CRISPR or related signaling molecules, such as SMADs, may enhance therapeutic outcomes [121]. Modifying genes in the β‐catenin/Wnt pathway may improve treatment effectiveness and reduce the expression of mesenchymal markers. Preclinical studies have suggested that inhibition of β‐catenin can enhance radiosensitivity and prevent EMT [69]. CRISPR components face challenges before they can be used in tumor cells for therapeutic applications. Using CRISPR/Cas9 to target the genetic factors involved in EMT could enhance treatment outcomes, particularly drug resistance and radiosensitivity [122]. Further research is essential to overcome these challenges and fully harness the potential of innovative therapies.

### 7.3 Immunotherapy

Immunotherapy is a comprehensive and innovative treatment for cancer and radiation‐induced injuries that targets EMT. However, this approach is less effective because EMT promotes immune evasion [112]. EMT cells may downregulate MHC molecules, thereby reducing T‐lymphocyte identification. Mesenchymal tumor cells produce higher levels of immune checkpoint proteins such as PD‐L1 (programmed death‐ligand 1), which restricts T‐cell activity [123]. Immunotherapy, which combines immune checkpoint inhibitors (ICIs) with EMT‐reversing drugs, may be more effective in targeting the EMT program. Cancers can become more susceptible to ICIs by targeting pathways that promote EMT, such as TGF‐β and Wnt/β‐catenin [124]. Hepatocyte growth factor (HGF) and its receptor c‐Met inhibitors increase antitumor immunity by reversing EMT in various malignancies, notably hepatocellular carcinomas. ICIs have revolutionized cancer treatment; however, PD‐1/PD‐L1 suppression and EMT often act against them. Research indicates that PD‐1 inhibition may be ineffective for mesenchymal tumors, whereas treatments targeting PD‐L1 and EMT pathways show inhibitory effects [125]. CTLA‐4 and PD‐1 inhibitors may reduce mesenchymal immune suppression when combined with EMT therapy [123]. AXL is upregulated during EMT and is associated with immune evasion. Owing to AXL inhibition, the tumor microenvironment may become more susceptible to ICIs [126]. EMT during immune evasion can be reduced by altering the tumor microenvironment using cytokines and chemokines that attract immune cells. By addressing the immune evasion mechanisms of EMT, this strategy could enhance the clinical outcomes in patients with cancer. Further research is required to optimize these techniques for clinical use.

### 7.4 Targeting Signaling Pathways

Pirfenidone functions as an anticancer agent, especially in lung cancer models, by inhibiting tumor growth and disrupting pathways linked to EMT [127]. Nintedanib is an FDA‐approved tyrosine kinase inhibitor used to treat idiopathic pulmonary fibrosis (IPF). It alters the pathways linked to fibrosis, including those associated with PDGF, FGF, and VEGF signaling. This multi‐target approach targets signals that may reduce the fibrotic response associated with EMT [128]. The Wnt/β‐catenin pathway significantly affects EMT regulation and cancer development. Activation of this pathway increases β‐catenin nuclear translocation, enhances EMT‐related gene expression, and promotes metastasis and radioresistance [26]. Inhibitory drugs such as FH535 can reverse EMT and improve radiosensitivity in malignancies, including esophageal and colorectal tumors, by inhibiting β‐catenin [129]. Notch signaling regulates EMT and cell differentiation, promoting EMT transcription factors and tumor growth. Therapeutic strategies, such as γ‐secretase inhibitors, can suppress Notch signaling, reverse EMT, and improve sensitivity to chemotherapy and RT [130]. The PI3K/Akt pathway regulates cell survival and growth. This pathway activates mesenchymal transition transcription factors to induce EMT. ACT001 and other PI3K/Akt inhibitors have been used to reverse EMT and boost cisplatin effectiveness [131]. Several mechanisms regulate the EMT. EMT is inhibited by S1P pathway antagonists that modulate STAT3 signaling, which is linked to acquired radioresistance [132].

### 7.5 Anti‐Fibrotic Drug‐Based Therapy

Pirfenidone is a medication that helps manage IPF by inhibiting the formation of scar tissue. The main contributor to EMT is the inhibition of TGF‐β signaling [133]. Pirfenidone may modify the fibrotic and inflammatory processes, thereby influencing the mechanisms involved in EMT. Animal research has demonstrated that pirfenidone may serve as an anticancer drug, especially in lung cancer models, by impeding tumor growth via its interaction with pathways associated with EMT [134]. Nintedanib is an FDA‐approved tyrosine kinase inhibitor that is used to treat IPF. It alters pathways relevant to fibrosis, including those linked to VEGF, PDGF, and FGF signaling. This multi‐target strategy may mitigate the fibrotic response associated with EMT [135]. Disulfiram has also shown promise in cancer therapy. It inhibits EMT and metastasis by affecting the NF‐κB and TGF‐β signaling pathways. Its potential effectiveness against hepatocellular carcinoma stems from its ability to tackle issues related to EMT [136]. Ongoing clinical studies are investigating its effectiveness in treating various cancers, including its ability to enhance the susceptibility of cancer cells to chemotherapeutic agents. The concomitant use of pirfenidone or nintedanib with chemotherapy may enhance the therapeutic efficacy against both fibrosis and cancer. Concentrating on anti‐fibrotic pathways is an approach that can improve the difficulties of EMT in cancer and radiation treatment [137]. Recent studies have shown improved therapeutic efficacy for patients with advanced metastatic cancer and associated fibrotic alterations by combining novel compounds, such as disulfiram, with existing anti‐fibrotic medicines, such as pirfenidone and nintedanib [127].

### 7.6 Natural Compounds

Research and treatment have focused on natural compounds to inhibit EMT, improve chemosensitivity, and limit metastasis in cancer therapies, including RT. Curcumin, resveratrol, and gentiopicroside are key drivers of EMT and TGF‐β signaling [138]. Sulforaphane and luteolin inhibit β‐catenin signaling, a process associated with the promotion of EMT. This inhibition may assist cancer cells in recovering their epithelial characteristics and transcription factors that promote the inhibition of SNAIL and TWIST during EMT [139]. The expression of EMT transcription factors, such as SNAIL and TWIST, which are crucial for the induction of the mesenchymal phenotype, was downregulated by gedunin and honokiol [140]. Methyl gallate downregulates mesenchymal markers such as vimentin while simultaneously upregulating E‐cadherin. This dual action contributes to the reversal of EMT in cancer cells [141]. Curcumin, which is derived from turmeric, has been extensively studied for its anticancer properties. It inhibits multiple signaling pathways associated with EMT, such as TGF‐β and Wnt/β‐catenin. Curcumin enhances the chemosensitivity of resistant cancer cells by addressing EMT‐related alterations [142]. Resveratrol exhibits anti‐inflammatory and anticancer properties. It modifies several signaling pathways, such as TGF‐β, and reduces the synthesis of EMT transcription factors, consequently inhibiting metastasis [143]. Apogossypolone (ApoG2), a gossypol derivative, inhibits cervical cancer cell invasion and proliferation by blocking the EMT process [144]. Methyl gallate compounds have shown potential in hepatocellular carcinoma and inhibit cell proliferation and migration while regulating MMPs and the AMPK/NF‐κB pathway, effectively reversing EMT [145]. Thus, they represent a potential class of drugs that can target EMT for cancer treatment. As they can alter the transcription factors and signaling pathways of EMT, they provide a diverse strategy for overcoming challenges such as drug resistance and metastasis.

### 7.7 Nanoparticles

These nanoparticles use the Enhanced Permeability and Retention (EPR) phenomenon to passively target tumors through their vasculature, enhancing drug concentration at tumor locations while avoiding organ damage [146]. Gold nanoparticles have shown potential in reversing the mesenchymal phenotype in various cancer types by inhibiting EMT markers, such as vimentin and N‐cadherin, while enhancing E‐cadherin expression [147]. Silicon dioxide and titanium dioxide nanoparticles downregulate the Smad2/3 signaling pathway, which is crucial for EMT activation, thereby diminishing TGF‐β‐mediated EMT. This circulation method delivers therapeutic agents to acidic environments or tumor tissues with high ROS levels [148]. The development of pH‐sensitive and redox‐responsive nanoparticles that react to tumor microenvironment cues allows for this. Recent studies have focused on the co‐delivery of multiple therapeutic agents using nanoparticles, siRNAs, and chemotherapeutic drugs. siRNAs targeting Snail and Twist can be delivered via nanoparticles and conventional chemotherapeutics, such as paclitaxel. This multi‐target strategy has shown promising results in preclinical animal studies for inhibiting tumor growth and metastasis [149]. Nanoparticles can improve photothermal or photodynamic therapy for EMT and pharmaceutical delivery. Many cancer types exhibit reduced invasion and metastasis with nanoparticle composition. Cold plasma treatment with PEG‐coated gold nanoparticles altered EMT markers in glioblastoma mice, reducing tumor growth [150]. A recent study suggested that targeting EMT may offer novel cancer treatments. Nanoparticles can enable combination therapy, directly suppress EMT, and increase drug delivery in aggressive cancers with metastases and treatment resistance. Further studies are needed to properly understand these tactics and address the present difficulties.

### 7.8 Combination Therapies

To enhance therapeutic efficacy, current oncology and radiology drugs targeting EMT employ combination therapy to address the mechanisms of EMT and drug resistance. Tumors develop resistance to radiation and chemotherapy via EMT, which promotes metastasis [151]. Recent studies have shown that TGF‐β inhibitors can reverse EMT, improve tumor radiosensitivity when combined with radiation therapy, and enhance treatment outcomes by preventing EMT. Additionally, many radioresistant cancers exhibit an active PI3K/Akt/mTOR signaling pathway. Combined with dual inhibitors such as BEZ235, radiation therapy can reduce EMT markers and enhance apoptosis in radioresistant prostate cancer cells [152]. The combination of chemotherapy and targeted therapy with EMT‐targeted drugs may enhance the efficacy of cancer treatment. Matthew et al. found that radiation treatment with T‐DM1 treated breast cancer cells. Locoregional therapy offers a good safety profile; however, radionecrosis is a risk [153]. Mesenchymal phenotypes are linked to immunosuppression, and ICIs, such as PD‐1/PD‐L1 inhibitors, can potentially boost antitumor immunity when combined with agents that reverse EMT. Targeting TGF‐β and PD‐L1 simultaneously may improve therapeutic responses in cancers marked by significant EMT [154]. Personalized therapies are essential because of the unpredictable nature of tumor biology and the diversity of cancers. Although combination therapies targeting EMT are promising, the development of biomarkers for predicting responses is crucial. Combination pharmacotherapy can effectively address the challenges associated with the EMT. Future research should aim to improve treatment efficacy through targeted therapies that disrupt the signaling pathways of RT and chemotherapy.

### 7.9 Epigenetics

During EMT, epigenetic changes primarily regulate gene expression, including DNA methylation, histone modification, and noncoding RNA production. Alterations in histone acetylation and methylation can affect the expression of EMT‐associated genes. An excess of histone acetyltransferases (HATs) enhances the expression of epithelial markers, whereas a deficiency in histone deacetylases is often linked to the EMT process [155]. Noncoding RNAs, particularly microRNAs, are key regulators of EMT. MiR‐200 targets transcription factors such as ZEB1 and ZEB2, and its reexpression can reduce EMT and increase chemotherapy sensitivity. Moreover, long noncoding RNAs (lncRNAs) promote EMT by affecting the expression of related genes, and targeting these lncRNAs may offer a novel therapeutic strategy [156]. Histone deacetylase inhibitors, such as vorinostat and romidepsin, can correct histone modifications during EMT. This mechanism improves the effectiveness of RT and chemotherapy in eradicating cancer cells, resulting in a resurgence of epithelial markers [114]. Decitabine and azacitidine are inhibitors of DNA methyltransferase. These pharmaceuticals can demethylate DNA and reactivate previously repressed tumor suppressor genes, which are crucial for preserving epithelial traits [157]. Combining regular chemotherapy with HDAC inhibitors enhances their effectiveness against various cancers by overcoming drug resistance associated with EMT. DNMT inhibitors may improve radiosensitivity and outcomes in radiation‐resistant cancers by inhibiting radiation‐induced EMT [158]. Recent studies have focused on the signaling systems that regulate epigenetic changes and EMT. Inhibition of TGF‐β may impede EMT and its associated epigenetic modifications. Epigenetic regulators and TGF‐β inhibitors may improve the effectiveness of therapies for cancer cells with increased EMT levels [159]. Epigenetic approaches that impede EMT have been shown to improve therapeutic outcomes in cancer.

### 7.10 Metabolic Targeting

Cancer cells rely heavily on glycolysis (Warburg effect) to produce energy, particularly under hypoxic conditions. Targeting glycolysis inhibits EMT and reduces resistance to treatment. As glycolysis reduces ATP availability and alters cellular metabolism, it can reduce the expression of EMT markers and genes. Similar to glucose, 2‐deoxy‐d‐glucose (2‐DG) inhibits glycolysis, reduces EMT, and increases radiosensitivity [160]. 3‐Bromopyruvate inhibits glycolysis, reduces energy production, and reverses EMT in cancer cells. Glutamine is essential for cancer cell growth because it supplies carbon and nitrogen for biosynthesis. Targeting glutamine metabolism reduces EMT‐driven resistance [161]. Glutaminase inhibitors block the TCA cycle by inhibiting the conversion of glutamine to glutamate, thereby reducing the expression of EMT markers. The potent glutaminase inhibitor CB‐839 has been shown to decrease EMT and enhance radiosensitivity in lung and breast cancer cells [162]. Trimetazidine, which blocks fatty acid oxidation, has the potential to inhibit EMT in prostate and liver cancer [163]. Metformin is an antidiabetic drug that inhibits mitochondrial Complex I, reduces EMT, and improves radiosensitivity by suppressing NF‐κB and PI3K/Akt signaling. Phenformin is a mitochondrial metabolism inhibitor similar to metformin that reverses EMT and reduces metastatic capacity [164]. However, excessive ROS can lead to oxidative stress, disrupting EMT signaling pathways such as NF‐κB and PI3K/Akt. Auranofin increases ROS levels and effectively reduces EMT markers, which helps inhibit cancer cell invasiveness [165]. In breast and prostate cancers, an inhibitor of fatty acid synthase reduces epithelial simvastatin statin, which inhibits cholesterol synthesis, reduces EMT markers, and improves radiosensitivity in several cancers [166]. Asparaginase reduces asparagine availability and EMT in leukemia and solid tumors. PHGDH inhibitors target serine biosynthesis, which is crucial for EMT and cell proliferation in certain cancer types [167]. Lactate transporters help cancer cells manage high lactate production associated with glycolysis. Inhibition of these transporters can disrupt the acidic tumor microenvironment and promote EMT. Blocking lactate transporters, such as MCT1, reduces lactate buildup, lowers pH, and downregulates EMT [168]. AZD3965 initiates MCT1 inhibition, which reduces EMT markers and improves the RT response by limiting lactate transport in cancer cells [169]. Metabolic targeting of EMT pathways represents a novel strategy in cancer therapy that helps overcome drug and radiation resistance, reduces metastasis, and enhances treatment efficacy.

## 8 Toxicological Considerations in EMT‐Targeted Therapies Combined With Radiation

EMT‐targeted therapies enhance radiosensitivity and inhibit tumor progression. However, their combination with RT poses a toxicological challenge. A primary concern involves off‐target effects, given that EMT pathways such as TGF‐β, Notch, and Wnt play important roles in normal tissue regeneration and immune regulation [170]. Inhibition of these pathways can lead to tissue injury, fibrosis, and delayed healing. Inhibition of the TGF‐β pathway is critical for EMT to resolve radiation‐induced damage. In preclinical mice, calunisertib and vactosertib combined with thoracic or lumbar radiation exacerbated pulmonary fibrosis, mucositis, and gastrointestinal inflammation [171]. Murine studies have indicated that TGF‐β inhibition after thoracic radiation causes significant alveolar damage, persistent inflammation, and reduced survival (Clinicaltrials.gov; NCT01373164). Patients receiving combination therapy exhibited greater fatigue, gastrointestinal irritation, and increased liver enzyme levels. Notch signaling inhibitors, such as MK‐0752 and RO4929097, can cause hematological toxicity and mucosal thinning. Together with radiation, they cause intestinal crypt cell death and ulceration [172]. The Wnt pathway modulator LGK974 can reverse EMT but can cause enteropathy, baldness, and skeletal fragility in rapidly renewing tissues, thereby worsening RT‐induced damage [173]. Immunologically related toxicity can affect immunological control through T‐cell fatigue and macrophage polarization via EMT pathways. Dual pathway inhibitors, such as bintrafusp alfa, can produce immunologically related adverse effects such as colitis, dermatitis, and pneumonitis, especially when administered with radiation, which increases inflammation and immune suppression [174]. The Phase I NCT02517398 study found that 31% of participants had Grade 3/4 toxicity. EMT helps repair mucosal and vascular tissues after radiation‐induced inflammation. Inhibitors may slow healing, increase bleeding, and cause late toxicity [175]. To minimize these risks, strategies including biomarker‐guided patient selection, E‐cadherin, vimentin, EMT‐associated miRNAs, optimized fractionation, spatially precise RT using IMRT, and tumor‐specific delivery systems for nanoparticles and exosomes can be used [176]. Future trials should incorporate thorough safety monitoring that combines EMT suppression with the maintenance of normal tissue integrity, thereby enhancing the therapeutic index of RT.

## 9 EMT and Clinical Relevance

### 9.1 EMT Biomarkers for Diagnostic and Prognostic Use in Clinical Samples

EMT biomarkers link tumor biology to clinical practice, enhancing diagnosis, prognosis, and treatment planning in patients with cancer. In breast cancer, colon cancer, and HNSCC, decreased E‐cadherin expression is associated with tumor differentiation, invasion, lymph node metastasis, and resistance to radiation. Low E‐cadherin levels identified by immunohistochemistry are associated with poor progression‐free survival (PFS) and overall survival (OS), and some tumor grading systems now consider E‐cadherin loss as a negative prognostic sign [177]. However, mesenchymal markers, including vimentin, N‐cadherin, and fibronectin, are persistently elevated throughout EMT, indicating invasive and metastatic tendencies. Increased vimentin expression is associated with poor outcomes and low radiosensitivity in glioblastoma, NSCLC, and esophageal cancer. N‐cadherin overexpression, or the “cadherin switch,” is common in prostate and bladder cancers and is linked to bone metastases and therapy resistance [178]. EMT transcriptional regulators, including Snail, Slug, Twist, ZEB1, and ZEB2, have also been identified as potential prognostic biomarkers. Snail overexpression in hepatocellular carcinoma and pancreatic cancer is associated with chemoresistance and poor survival. In cervical and breast cancers, ZEB1 expression is associated with distant metastases and recurrence after radiation [179]. Protein indicators and miRNAs regulate EMT and are potential noninvasive biomarkers. MiR‐200a/b/c, miR‐141, and miR‐429 target ZEB1/2 to preserve the epithelial phenotype and are downregulated in metastatic tumors, making them good predictors of EMT status [180]. miR‐21 is observed in various malignancies and supports EMT by inhibiting tumor suppressor genes, including PTEN and PDCD4 [30]. In the NCT04572451 clinical trial for head and neck cancer, plasma or serum miRNA levels were tested as liquid biopsy indicators for real‐time EMT monitoring and diagnosis of treatment resistance [181]. Tumor‐derived exosomes containing mesenchymal markers and specific RNAs, such as MALAT1 and HOTAIR, can be used to dynamically and noninvasively monitor EMT development. In lung adenocarcinoma, high levels of exosomal miR‐23a promote EMT and early metastasis. Exosomes from blood or saliva facilitate tumor surveillance in patients undergoing RT or chemotherapy. Incorporating EMT markers into multi‐omics systems improves precision oncology tools [182]. Techniques such as single‐cell RNA sequencing, digital droplet PCR, and mass cytometry can be used to assess EMT heterogeneity and distinguish various EMT stages with different therapeutic implications. These advances have shifted the focus of EMT biomarkers from research to practical applications, including early diagnosis, patient stratification, and MRD monitoring. E‐cadherin, vimentin, Snail, ZEB1, and miR‐200 family members are biomarkers that play both therapeutic and diagnostic roles [183]. Their use in biopsies and liquid biopsies is critical for next‐generation oncology diagnostics to improve the accuracy and efficacy of EMT‐targeted and radiation‐based therapies.

### 9.2 Clinical Implications of EMT‐Targeted Therapies in Precision Oncology

EMT allows tumor cells to transition from an epithelial to a mesenchymal state, thereby increasing their ability to invade and evade the immune system. Targeting EMT has become a promising treatment strategy, particularly when combined with RT. Inhibitors targeting the Wnt/β‐catenin, Notch, and TGF‐β pathways, such as ICG‐001, RO4929097, and vactosertib, have been developed. However, their clinical application is hampered by the essential role of EMT in healing processes, such as tumor heterogeneity, off‐target effects, and the risk of immunosuppression due to TGF‐β inhibition [184]. Information is provided on the use of these treatments in combination with other treatments to reduce adverse effects. Combining EMT‐targeted agents with ICIs, anti‐PD‐1/PD‐L1, or radiation holds promise in overcoming these challenges. Pintrafusp alpha is a fusion protein targeting TGF‐β and PD‐L1, which shows promise in reactivating the immune system and reversing EMT in solid tumors. Preclinical studies have indicated that epigenetic modulators, such as HDAC and EZH2 inhibitors, can reduce cancer stem‐like properties and make EMT‐positive tumors more sensitive to radiation therapy [185]. Real‐time monitoring of EMT biomarkers, such as tumor cell turnover and exosomal miRNAs, is essential for guiding adaptive therapeutic strategies. Advanced single‐cell RNA sequencing techniques have provided insights into EMT dynamics, aiding the development of personalized treatment plans [186]. Although EMT‐targeted therapies can potentially improve radiation efficacy and reduce resistance, careful patient selection and a multidisciplinary approach are essential for successful implementation. The integration of precision oncology and biomarker‐based strategies will help to ensure the effectiveness and safety of these treatments. However, their efficacy in cancer patients requires continuous clinical trials to assess safety.

### 9.3 Clinical Outcomes and Challenges of EMT Research

Loss of epithelial markers, such as E‐cadherin, and gain of mesenchymal markers, such as vimentin and N‐cadherin, are associated with poor prognosis and reduced survival in HNSCC, NSCLC, and breast cancer. EMT is associated with tumor progression, metastasis, and resistance to treatment. In NSCLC, high TGF‐β1 levels are associated with a 40% increase in recurrence after radiation [187]. Targeting radiation‐induced EMT is essential for improving the outcomes of resistant cancers. Clinical trials are investigating the use of EMT‐inhibiting agents to sensitize tumors to radiation. TGF‐β inhibitors, such as galunisertib, have shown a 30% increase in tumor regression in pancreatic and head and neck cancers [188]. It inhibits Wnt/β‐catenin signaling, reducing CSCs and EMT after radiation in pancreatic and colorectal cancers [189]. Notch signaling, which is important for cellular plasticity and EMT, is blocked by gamma‐surfactant inhibitors such as DAPT, downregulating EMT transcription factors and improving RT responses in breast cancer and glioblastoma [190]. The PI3K/AKT/mTOR pathway, associated with MT‐mediated resistance, can be targeted with everolimus to reduce mesenchymal markers and delay tumor recurrence in HNSCC, NSCLC, and renal cell cancer [191]. Tumors can develop resistance mechanisms, necessitating the use of multi‐targeted therapeutic approaches. Integrated approaches, including transcriptomics, proteomics, and epigenomics, can help identify responsive patient populations and novel therapeutic targets. EMT‐driven tumors are often resistant to checkpoint blockade therapies; therefore, immunotherapy must address immune evasion associated with EMT. Combining immunotherapy with EMT‐targeted agents may improve treatment outcomes for refractory cancers, enhance the efficacy of RT, reduce metastasis, and increase patient survival rates.

## 10 Future Directions and Emerging Research in EMT Modulation: Advancing Personalized Therapeutic Strategies

Targeting EMT in cancer therapy is challenging because these cells exhibit stemness, immune evasion, and survival advantages over other cells. The diversity and rapidity of these species make their identification challenging, necessitating high‐resolution diagnostics. New drugs, such as bintrafusp alfa and CB‐103, have been developed to reduce toxicity. Therapeutic targeting is guided by EMT mapping and biomarker identification [192]. Elevated *N*‐hexanoyl glycine and β‐thymidine levels in radiation‐induced EMT may be noninvasive markers for metabolic profiling in blood or urine [193]. The Human Tumor Atlas Network (HTAN) and The Cancer Genome Atlas (TCGA) are platforms that combine EMT gene signatures to predict recurrence and radiation sensitivity [194]. Advanced tools, such as single‐cell RNA sequencing, spatial transcriptomics, and patient‐derived organoids, provide new insights into the spatiotemporal dynamics of EMT, supporting the development of adaptive, personalized therapeutic strategies. Because EMT occurs across a spectrum, including epithelial, mesenchymal, and hybrid stages, multi‐omics profiling, including transcriptomics, proteomics, and epigenomics, is critical. Biomarkers such as E‐cadherin, vimentin, ZEB1, SNAIL, and miR‐200 family members enable real‐time monitoring of EMT [195]. Liquid biopsies that capture circulating tumor cells or exosomal miRNAs, such as miR‐200c, miR‐155, and miR‐210, offer minimally invasive tools for dynamic assessment [196]. Notably, exosomal miR‐200 is currently being evaluated as a marker of radiation response in NSCLC. New genomic technologies, such as CRISPR‐based functional screens, have identified new EMT regulators of SPDEF and RBMS3, which modify hybrid EMT phenotypes linked to relapse [197]. Epigenetic factors, including EZH2, DNMT3A, and HDAC6, modify chromatin accessibility in EMT drivers, such as Snail and Twist, supporting the results of romidepsin and tazemetostat clinical trials [198]. Research can develop a simulation of radiation‐induced EMT in real time while conserving tumor microenvironment diversity using personalized 3D organoids, tumor‐on‐a‐chip platforms, and single‐cell RNA sequencing. These techniques allow drug screening and detection of partial EMT phases in resistant cancers [199]. A recent study explored the use of Eltrombopag as a potential mitogen stimulant, angiogenesis promoter, and therapeutic radioprotectant through TPO‐R activation. The recovery of normal tissue exposed to ionizing radiation helps minimize the unintended side effects of tissue damage [200]. Future approaches are shifting toward systems biology and integrating multi‐omics, imaging, and AI‐driven analytics for precise EMT‐based diagnosis and personalized treatment. Large‐scale projects such as the HTAN and Dependency Map have accelerated EMT discovery across tumor types. These innovations represent a transformative shift in the management of EMT‐driven cancer resistance, enabling highly individualized and adaptive therapeutic strategies with the potential to significantly improve the outcomes of patients with aggressive tumors.

## 11 Conclusion

Various studies have shown that EMT contributes to fibrosis in both normal and radiation‐induced cancer conditions. EMT has significantly influenced therapeutic strategies over the past decade and plays a critical role in cancer progression, spread, and resistance to therapy. EMT has led to the development of therapeutic techniques for inhibiting or reversing this process in cancer patients. These strategies include inducing mesenchymal–epithelial transition (MET) to reduce invasiveness and improve sensitivity to chemotherapy. In addition, small drugs and multi‐omics approaches targeting pathways such as TGF‐β, Wnt/β‐catenin, ERK, and Notch are important. However, implementing these findings is challenging because of the limited efficacy observed in clinical trials. EMT plays a significant role in the development of drug resistance, particularly in chemotherapy, targeted therapy, and immunotherapy.

Recent research and clinical trials have investigated the effectiveness of combination treatments focusing on EMT and conventional cancer drugs. This approach aims to improve the efficiency of therapy and overcome possible resistance that may arise. EMT enables cancer cells to evade the immune system and is less effective than immunotherapy. Various therapeutic approaches have been explored for the treatment of different types of cancer, and these methods have proven more effective than older treatments. Therefore, we focused on targeting these pathways and presented ongoing and completed clinical trials on this website (https://clinicaltrials.gov/). Early‐stage clinical trials have begun using EMT biomarkers to classify individuals for targeted therapies. In addition, EMT may occur not only in cancer cells but also in healthy tissues because of the unintended consequences of cancer treatment, such as radiation and chemotherapy. Furthermore, naturally derived polyphenols have been shown to mitigate and inhibit EMT in cancer cells.

Extensive research has been conducted to develop preventive strategies, including the use of anti‐fibrotic drugs, to reduce therapy‐induced EMT in healthy tissues. These adverse effects significantly compromise the patient's quality of life and therapeutic outcomes. Future research should focus on developing combination therapies that target EMT, along with conventional treatments, to minimize metastasis, enhance drug resistance, and protect healthy tissues. An increasing number of patients are expected to utilize targeted therapeutic approaches tailored to their EMT profiles, leading to less harmful and more effective treatment options for cancer patients in the future. Although subsequent EMT in cancer therapy shows promise in improving clinical outcomes, several challenges remain to be addressed.

## Author Contributions

R.Su. and R.Sh. conceptualized this study. D.S., R.Su., and R.Sh. acquired the resources, curated the data, and drafted the original manuscript. R.B., A.C., J.K., D.M.G., and R.S. contributed to resource provision, review, and editing of the manuscript. All authors approved the final version after their contributions and critical evaluations.

## Ethics Statement

The authors have nothing to report.

## Conflicts of Interest

Dr. Dinesh Murugan Girija is an employee of Vopec Pharmaceuticals Pvt. Ltd., Chennai, India. The other authors declare no conflicts of interest.

## Data Availability

All data relevant to this study have been incorporated into this article.
